# Supplementary material for: Targeted activation of midbrain neurons restores locomotor function in mouse models of parkinsonism
Source: Nat Commun. 2022 Jan 26;13:504. doi: 10.1038/s41467-022-28075-4 (PMC8791953; doi:10.1038/s41467-022-28075-4)
Supplement: Supplementary file 1 — Supplementary Information [file 41467_2022_28075_MOESM1_ESM.pdf]

Supplementary information

Targeted activation of midbrain neurons restores locomotor function in mouse models of parkinsonism

Débora Masini[1] and Ole Kiehn[1,2]*

[1]Department of Neuroscience, Faculty of Health and Medical Sciences, University of Copenhagen, Blegdamsvej 3B, 2200 Copenhagen N, Denmark and [2]Department of Neuroscience, Karolinska Institutet, Stockholm, Sweden

*Correspondence: Ole.Kiehn@sund.ku.dk

Contents

- **Supplementary figures 1 to 10**
- **Statistical Tables S1 to S7** (stats reported in order of text citation)
- **Table S8** contains information on the Mouse cohorts
- Legend description for Movies 1 and 2

Content pages

- Supplementary Fig.1 ------------------------- page 3
- Supplementary Fig.2 ------------------------- page 5
- Supplementary Fig.3 ------------------------- page 7
- Supplementary Fig.4 ------------------------- page 9
- Supplementary Fig.5 ------------------------- page 10
- Supplementary Fig.6 ------------------------- page 12
- Supplementary Fig.7 ------------------------- page 15
- Supplementary Fig.8 ------------------------- page 17
- Supplementary Fig.9 ------------------------- page 19
- Supplementary Fig.10 ------------------------- page 21

- Supplementary Table 1 ------------------------- page 23
- Supplementary Table 2 ------------------------- page 26
- Supplementary Table 3 ------------------------- page 27
- Supplementary Table 4 ------------------------- page 31
- Supplementary Table 5 ------------------------- page 33
- Supplementary Table 6 ------------------------- page 35
- Supplementary Table 7 ------------------------- page 37

- Supplementary Table 8 (mouse cohorts) -------- page 40

- Legend description for Movies 1 and 2 ----------page 41

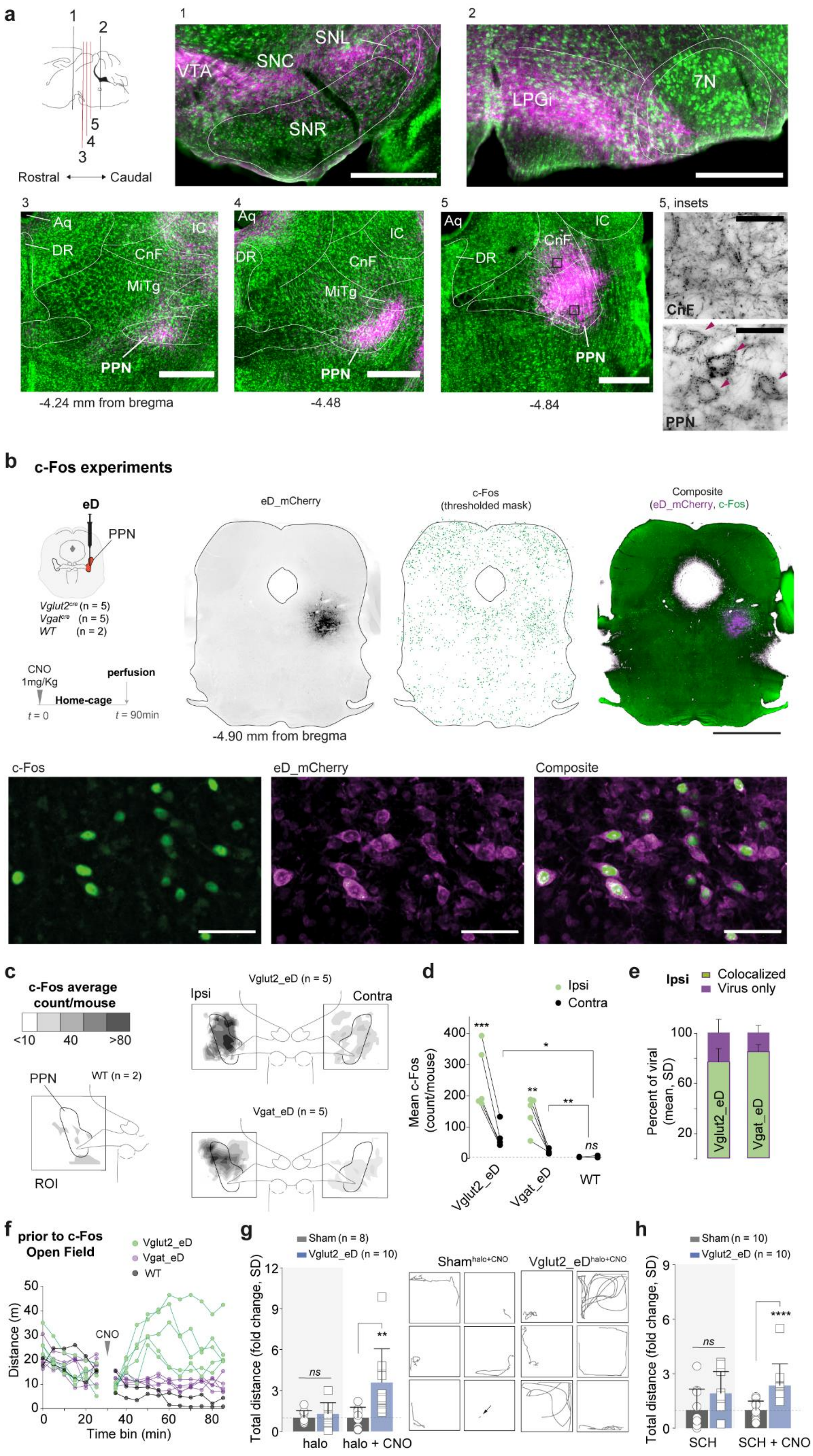
a
Rostral Caudal
VTA
SNC
SNL
SNR
LPGi
7N
Aq
DR
CnF
IC
MiTg
PPN
-4.24 mm from bregma
-4.48
-4.84
5, insets
b
c-Fos experiments
eD
PPN
eD_mCherry
c-Fos (thresholded mask)
Composite (eD_mCherry, c-Fos)
Vglut2cre (n = 5)
Vgatcre (n = 5)
WT (n = 2)
CNO 1mg/Kg
perfusion
Home-cage
t = 0
t = 90min
-4.90 mm from bregma
c-Fos
Composite
c
c-Fos average count/mouse
<10 40 >80
WT (n = 2)
ROI
Vglut2_eD (n = 5)
Vgat_eD (n = 5)
Ipsi
Contra
d
Mean c-Fos (count/mouse)
Vglut2_eD
Vgat_eD
WT
ns
e
Colocalized
Virus only
Percent of viral (mean, SD)
f
prior to c-Fos Open Field
Distance (m)
Time bin (min)
CNO
g
Sham (n = 8)
Vglut2_eD (n = 10)
Total distance (fold change, SD)
halo
halo + CNO
Shamhalo+CNO
Vglut2_eDhalo+CNO
h
Sham (n = 10)
Vglut2_eD (n = 10)
SCH
SCH + CNO

Supplementary Fig. 1 (S1)│ Supporting data for experiments with Vglut2_eD mice showing anatomical projections, caudal targeting, c-Fos induced expression upon CNO treatment and cumulative data in the Open Field.

(a) Microscope images of five representative coronal slices showing neurons in green (NeuroTrace) and DREADD-expressing $Vglut2^{+}$ cells stained in magenta. Upper panels show ascending (1) and descending (2) fiber projections of glutamatergic PPN neurons. Lower panels (3-5) show rostral to caudal cell distribution within the PPN boundaries with higher infections of neurons in the caudal than the rostral part. High magnification insets (black squares in panel 5) show cell bodies in PPN (arrows) and fibers within CnF (here only viral signal is shown). Scale 500µm, inset 25µm. Boundaries based on Mouse brain atlas[117].

(b) Experiment to confirm CNO induced activity of neurons expressing excitatory DREADDs ('eD'). Mice were injected with CNO (1mg/kg, ip) and perfused 90 min later. Sections were stained for c-Fos (green), a marker of cellular activation. Upper panel shows an example section from a $Vglut2^{cre}$ mouse injected unilaterally with eD (bregma -4.96mm). The injected hemisphere contains a high density of $c\text{-}Fos^{+}$ nuclei (scale 1mm). Lower panel shows a high magnification of the injected hemisphere with neurons expressing eD in magenta and $c\text{-}Fos^{+}$ nuclei in green (scale 50µm).

(c) Contour maps (from experiment in **b**, bregma -4.60mm) show the average number of $c\text{-}Fos^{+}$ nuclei found within an ROI (1.2mm side) centered over the PPN region. In WT mice, both hemispheres were treated as replicates prior to averaging between mice. For unilaterally injected mice, ipsi- and contralateral hemispheres are shown in separate. Note the high density of positive $c\text{-}Fos^{+}$ nuclei found within the boundaries of the PPN on ipsilateral (injected) hemispheres of $Vglut2^{cre}$ and $Vgat^{cre}$ mice (the two mouse lines used in this study).

(d) Quantification of $c\text{-}Fos^{+}$ nuclei in **c**. Connected lines show the average number of stained nuclei found per ROI in each subject. The ipsilateral hemisphere (green) shows a higher c-Fos count than the non-injected contralateral side (black) (Two-way RM ANOVA, hemisphere $F_{(1, 9)}=31.35$ with p=0.0003, report shows Sidak's ipsi vs contra. Comparing ipsi between groups: Brown-Forsythe and Welch ANOVA, B-F= $9.459_{(2, 4.283)}$ with p=0.0268; W=$16.56_{(2, 6.289)}$ with p=0.0031, report shows Dunnett's multiple comparison to WT group; Vglut2_eD p=0.0432, Vgat_eD p=0.0045).

(e) Colocalization of $c\text{-}Fos^{+}$ nuclei and viral mCherry expression (eD), performed on images with higher magnification (as in lower panel **b**). Only the ipsilateral hemisphere was sampled in Vglut2_eD and Vgat_eD mice. Neurons were divided into two categories: colocalized (green) or virus only (purple) (together=100%). In both genotypes colocalization is found in more than 75% of the total count while a minority of the eD-expressing neurons did not contain nuclear c-Fos (non-c-Fos: Vglut2_eD = 23±11%; Vgat_eD = 15±6% [mean±SD]).

(f) Prior to the c-Fos experiment (**b-e**) this cohort of mice was tested in one session of the Open Field. The distance traveled by each mouse before (25min) and after CNO (bins 35-85min) is shown. Note how, after CNO injection, both $Vglut2^{cre}$ and $Vgat^{cre}$ mice locomote more than the 2 WT mice that served as controls (black lines) (Brown-Forsythe and Welch ANOVA, B-F=$21.35_{(2, 4.085)}$ with p=0.00069; W=$119.6_{(2, 5.489)}$ with p<0.0001. Dunnett's multiple comparison to WT group; Vglut2_eD p=0.0088, Vgat_eD p=0.0002). These data indicate that mice used for c-Fos count show a similar locomotor profile as those reported in the main text.

(g) Left panel shows fold differences in total distance travelled in the Open Field. Data was normalized to Sham (grey) and Vglut2_eD (blue) mice after D2 receptor antagonism with haloperidol ('halo') and upon CNO treatment (two-tailed, t-test with Welch's correction, analysis done for each period separately). Right panel shows example traces for CNO-treated Sham and Vglut2_eD mice during the last 5 min of the test (minute 45 to 50). Six mice per group were randomly selected as examples.

(h) Fold differences in total distance travelled in the Open Field. Data was normalized to Sham (grey) and Vglut2_eD (blue) mice after D1 receptor antagonism with SCH23390 (SCH) and upon CNO treatment (two-tailed, t-test with Welch's correction, analysis done for each period separately).

Data are presented as mean ± SD. c-Fos mice: 5 $Vglut2\_eD^{CNO}$; 5 $Vgat\_eD^{CNO}$; 2 WT^{CNO}. Parkinsonian challenge mice: 8 $Sham^{halo+CNO}$; 10 $Vglut2\_eD^{halo+CNO}$; 10 $Sham^{SCH+CNO}$; 10 $Vglut2\_eD^{SCH+CNO}$. See also **Fig.1-3** and detailed stats in **Table S1**. **a(1-2)** VTA: ventral tegmental area, SNC: Substantia nigra, compact part, SNR: Substantia nigra, reticular part, SNL: Substantia nigra, lateral part, LPGi: lateral paragigantocellular nucleus, 7N: facial nucleus. **a(3-5)** Aq: aqueduct, DR: dorsal raphe, IC: inferior colliculus, CnF: cuneiform nucleus and precuneiform nucleus, MiTg: microcellular tegmental nucleus, PPN: pedunculopontine nucleus. Source data are provided as a Source Data file.

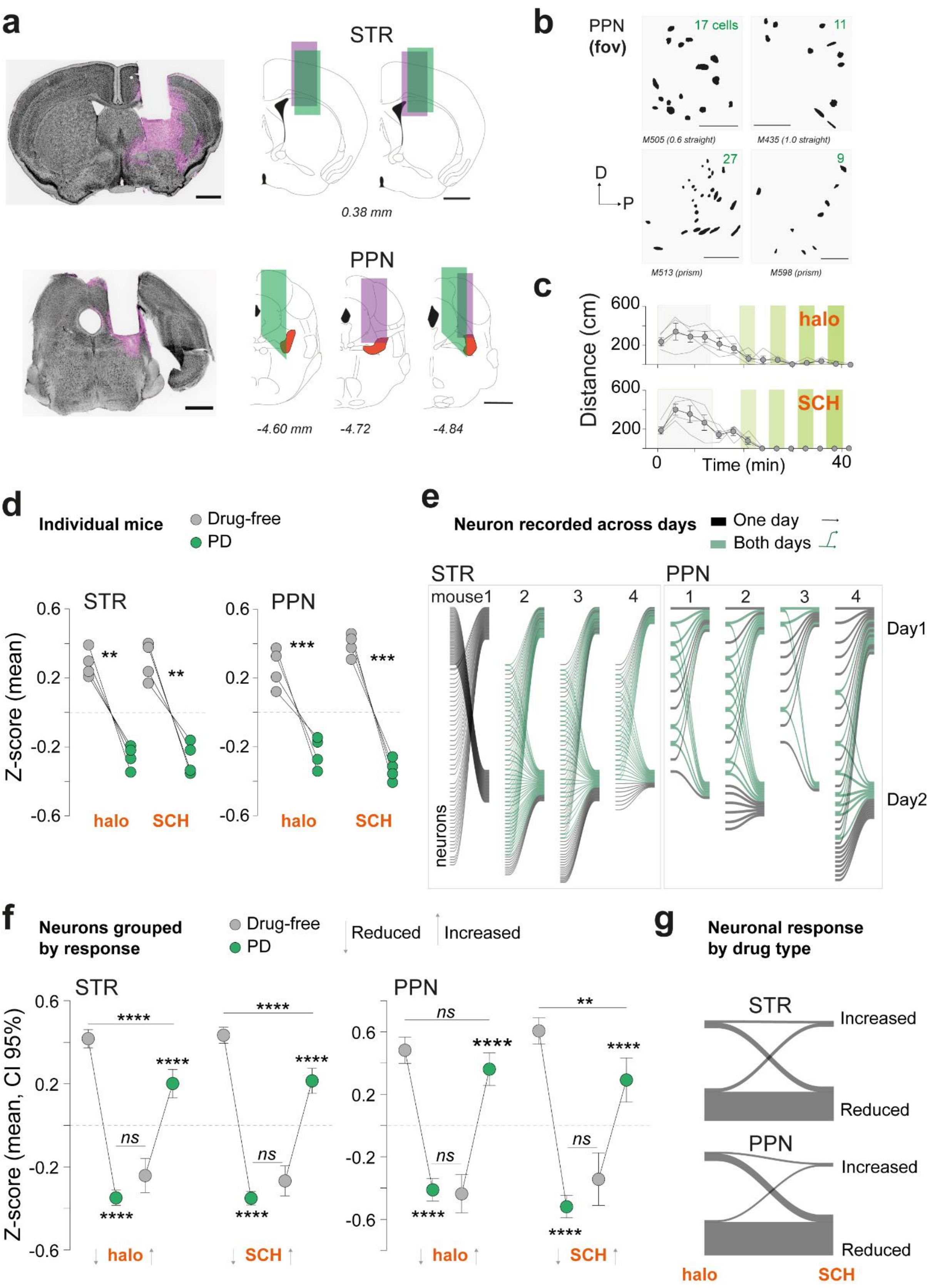
a
STR
0.38 mm
PPN
-4.60 mm
-4.72
-4.84
b
PPN
(fov)
17 cells
11
M505 (0.6 straight)
M435 (1.0 straight)
D
P
27
9
M513 (prism)
M598 (prism)
c
Distance (cm)
600
200
halo
SCH
0
Time (min)
40
d
Individual mice
Drug-free
PD
STR
PPN
Z-score (mean)
0.6
0.2
-0.2
-0.6
**
***
halo
SCH
e
Neuron recorded across days
One day
Both days
mouse1
2
3
4
1
neurons
Day1
Day2
f
Neurons grouped by response
Reduced
Increased
Z-score (mean, CI 95%)
****
ns
**
g
Neuronal response by drug type
STR
PPN
Increased
Reduced
halo
SCH

Supplementary Fig. 2 (S2) │ Supporting data related to Calcium imaging experiments with Drd1^{cre} (STR-GCamP6s) and Vglut2^{cre} (PPN-GCamP6s) cohorts of mice.

(a) Anatomical mapping of lens location. Left panels with example coronal slices. Right panels show schematic of lens location in all 8 experimental mice (STR, striatum; PPN, pedunculopontine nucleus; distance from bregma given under each coronal section [mm]). Scale 1mm and boundaries based on Mouse brain atlas[117] (n = 4 mice/group).

(b) Segmentation image of field of view (FOV) and PCA/ICA of identified glutamatergic neurons in PPN. Mouse ID and lens used are indicated under each image. Scale 100µm.

(c) PPN experiments. Behavioral quantification of distance moved (cm) throughout the test. Individuals represented by grey lines, with group mean ± SEM in black. Reduction of distance moved upon injection accounts for 74.13% of the total variance (Two-way RM ANOVA, time effect $F_{(13, 39)}$ =17.07 with p<0.0001, both sessions analyzed in conjoint).

(d) Population activity before (grey) and after (green) parkinsonian state induced by haloperidol or SCH injection. Connected lines show the average Z-score (in units of standard deviation [SD]) calculated per animal. Left panel shows STR and right panel shows PPN imaged mice (Two-way RM ANOVA, Interaction effect: STR, $F_{(1, 3)}$ = 0.03305 with p=0.8673 and PPN, $F_{(1, 3)}$ = 33.20 with p=0.0104, report shows Fisher's LSD multiple comparisons).

(e) Parallel plots showing for each mouse neurons that were recorded in one (black) or both experimental days (green, line bifurcates). Each plot represents one experimental mouse. On the left axis all neurons give rise to a line. If a neuron is recorded on both experimental days the line branches into two segments, connecting to experimental days 1 and 2. Mouse 1 (STR) had recordings performed with different focal planes between experimental sessions, therefore, all neurons are represented by single black lines.

(f) For each experimental day, neurons were classified according to their calcium dynamics response to the drug applied (**Fig.4g** and **5d**). Line graphs show average population Z-score for each classification/drug/genotype (units of SD ± 95% CI). This classification necessarily results in statistically significant changes on Z-score from drug-free to PD condition (Two-Way ANOVA, report shows Tukey's comparisons). Yet, the neurons increasing their activity do not reach the same Z-score as those which were active during baseline (Tukey's comparisons, with exception of PPN-halo with p=0.6612 [see *ns* in PPN panel top]).

(g) Parallel plots with axis scaling show that neurons that reduced their activity to halo can increase their activity under SCH and vice versa. On the left axis, neurons are distributed according to their response classification to haloperidol. Branching towards the right axis (SCH), neurons that swap response pattern create a cross-shaped path. Line thickness represents the proportion within all neurons of each response type (resulting proportions are reported in **Fig.4g** and **5d**).

See also **Fig.4-5** and detailed stats in **Table S2**. halo: haloperidol [D2-antagonist]; SCH: SCH23390 [D1-antagonist], PCA/ICA: combining Principal Component Analysis and Independent Component Analysis for spatial/temporal unmixing. Source data are provided as a Source Data file.

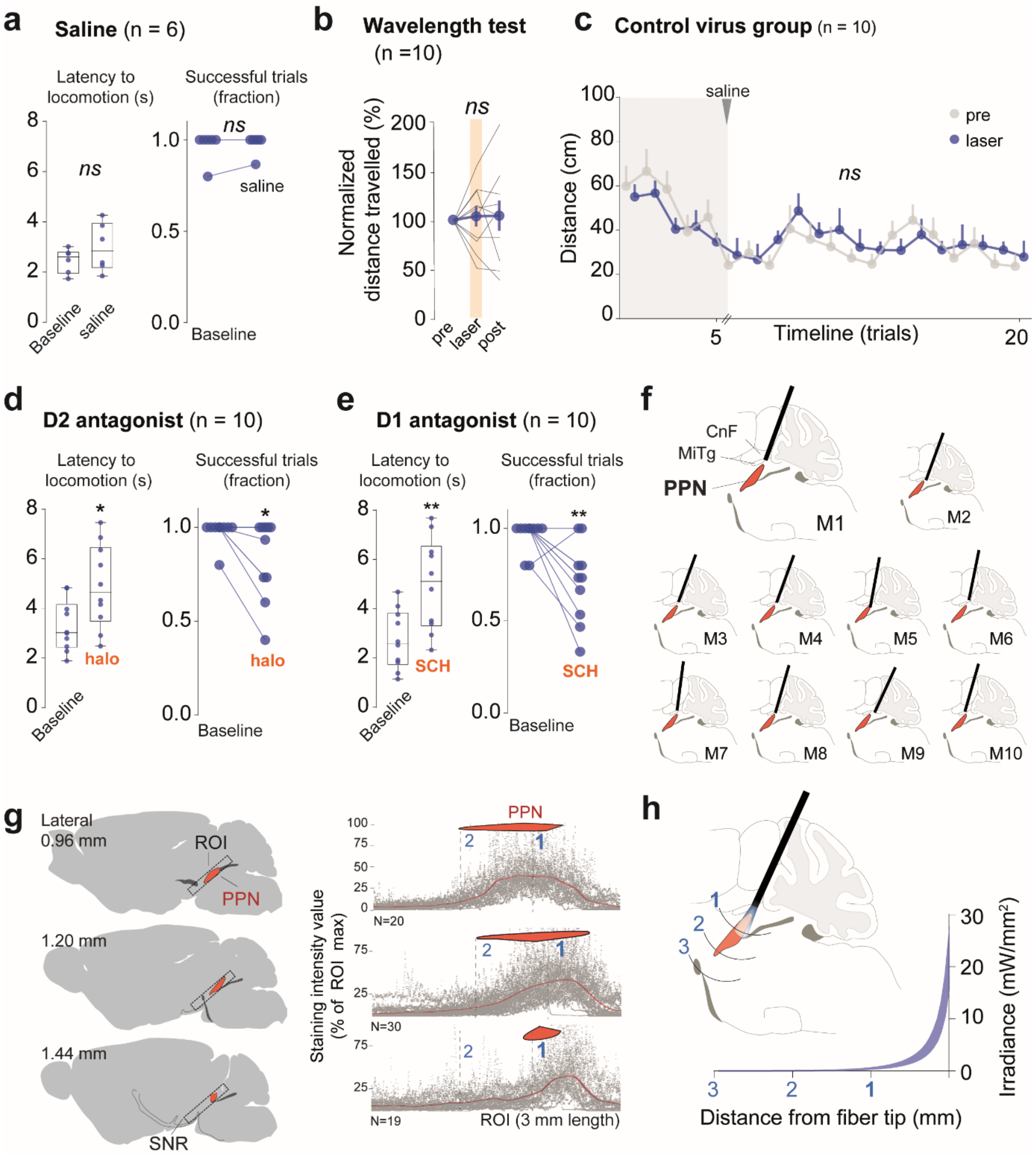

Supplementary Fig. 3 (S3)│ Supporting data for PPN optogenetic experiments with Vglut2_ChR2 mice showing motor latencies, fraction of successful trials and anatomical mapping.

(a) Saline injected mice. Min-to-max boxplots show latency (s) to initiate locomotion (Wilcoxon matched-pairs signed rank test, two-tailed) and connected lines show, for each mouse, the fraction of trials where light stimulation initiated a locomotor bout (paired t-test, two-tailed) before and after saline injection. Individual points (in blue) where calculated as the average value per animal during baseline (5 trials) and saline period (15 trials).

(b) Line graph with Normalized distance travelled (%) of 'pre', 'laser' and 'post' epochs in the Vglut2_ChR2 wavelength dependency test (593nm). Grey lines represent the average of eight trials for each mouse and the blue line represents group mean ± SEM (Friedman test -RM, followed by Dunn's comparison to 'pre', with graph report of multiple comparison).

(c) Timeline graph of a control group of mice injected with viruses that lacked ChR2 component. No effect of blue light-stimulation before (light-grey background) or after saline (mean ± SEM , Two-way RM ANOVA of laser condition vs trial timeline).

(d-e) Haloperidol ('halo', **d**) and SCH23390 ('SCH', **e**) experiments. Left panel, min-to-max boxplots show latency (s) to initiate locomotion. Individual points for average value per animal during baseline and drug period are shown (Wilcoxon matched pairs signed rank test, two-tailed). Right panels, connected lines, show the fraction of trials where light stimulation (473nm) initiated a locomotor bout before and after drug (Paired t-test, two-tailed).

(f) Anatomical fiber localization for the Vglut2_ChR2 mice included in the study. Sagittal mediolateral position = 1.20mm.

(g) Rostro to caudal quantification of staining intensity in Vglut2_ChR2 mice shown in **f**. Three sagittal segments were selected for analysis (0.96, 1.2 and 1.44mm lateral from bregma). Using a rectangular region of interest (ROI, 3mm length, 500µm height) placed over the PPN we extracted the staining intensity values for 23 sections/segment/mouse (e.g., lateral 0.96mm = 2sections/mouse, in 10 mice N=20 replicates). In blue, numbers indicate distance from fiber tip in **h**.

(h) Predicted irradiance (mW/mm^2) from optogenetic fiber in mammalian brain tissue. Laser power varied between 2-3.5mW (measured in the connector tip) and was maintained through experiments for each mouse. The figure shows the predicted irradiance levels within the mammalian brain tissue (see Methods). From the average end fiber position, three lines are drawn, each delimiting segments of 1mm distance from fiber tip. Plot shows decay in irradiance (shadow zone encompasses max and min laser power values) with nearly no light reaching beyond the caudal-most part of the PPN.

See also **Fig.6**, **S4** and **Movie 1**. Detailed stats in **Table S3**. halo: haloperidol [D2-antagonist]; SCH: SCH23390 [D1-antagonist]. In **f**, CnF: cuneiform nucleus and precuneiform nucleus, MiTg: microcellular tegmental nucleus, PPN: pedunculopontine nucleus. Source data are provided as a Source Data file.

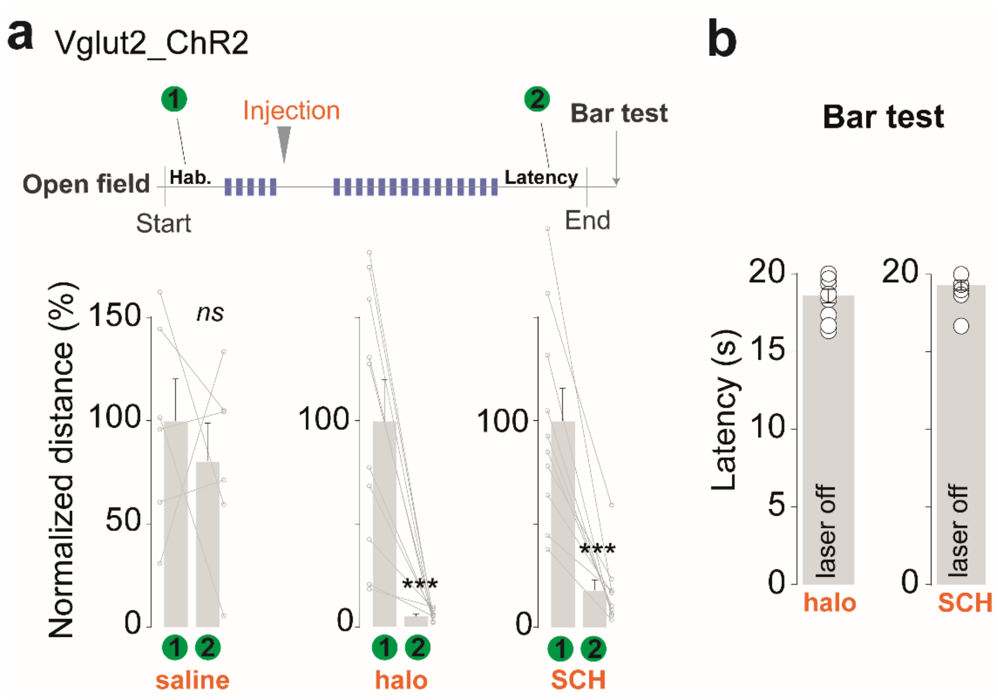

Supplementary Fig. 4 (S4)│ Confirmation of akinetic phenotype induced by dopamine antagonists in optogenetic experiments with Vglut2_ChR2 mice.

(a) Timeline for optogenetic experiments highlighting non-stimulus periods within the Open Field protocol used to confirm parkinsonian state development: Habituation ('Hab' ①) and latency to session end ('Latency' ②). Immediately after the Open Field session, mice performed the Bar test. Lower panel shows in sequence: saline, haloperidol and SCH injected experimental sessions. Distance travelled was normalized to habituation average (Wilcoxon matched-pairs, one-tailed).

(b) Bar graph shows impaired capacity to initiate movement in the Bar test for Vglut2_ChR2 mice treated with halo or SCH (time limit, 20s). Individual values represent average of 3 sequential trials per mouse.

Data are presented as mean ± SEM. Saline experiment = 6 mice; Halo = 10; SCH = 10. halo: haloperidol [D2-antagonist]; SCH: SCH23390 [D1-antagonist]. See also Fig.6, S3 and Movie 1. Detailed stats in Table S3. Source data are provided as a Source Data file.

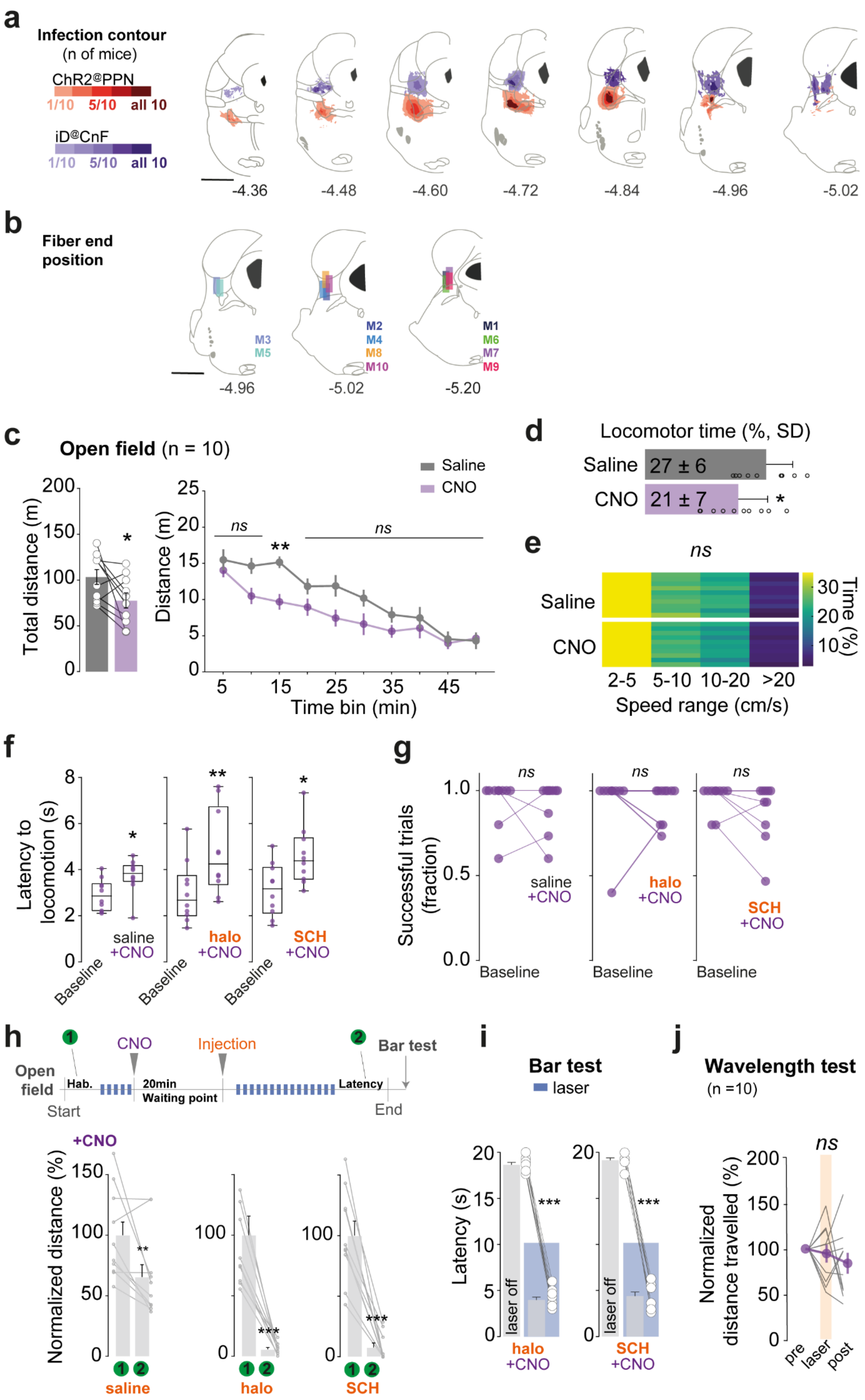
a
Infection contour
(n of mice)
ChR2@PPN
1/10 5/10 all 10
iD@CnF
1/10 5/10 all 10
-4.36 -4.48 -4.60 -4.72 -4.84 -4.96 -5.02
b
Fiber end position
M3 M5
M2 M4 M8 M10
M1 M6 M7 M9
-4.96 -5.02 -5.20
c
Open field (n = 10)
Total distance (m)
Distance (m)
Saline
CNO
ns
**
Time bin (min)
d
Locomotor time (%, SD)
Saline 27 ± 6
CNO 21 ± 7
e
ns
Saline
CNO
Time (%)
2-5 5-10 10-20 >20
Speed range (cm/s)
f
Latency to locomotion (s)
Baseline
saline +CNO
halo +CNO
SCH +CNO
g
Successful trials (fraction)
ns
Baseline
saline +CNO
halo +CNO
SCH +CNO
h
Open field
CNO
Injection
Bar test
Hab.
20min Waiting point
Latency
Start
End
+CNO
Normalized distance (%)
saline
halo
SCH
i
Bar test
laser
Latency (s)
laser off
halo +CNO
SCH +CNO
j
Wavelength test
(n =10)
Normalized distance travelled (%)
ns
pre laser post

Supplementary Fig. 5 (S5)│ Supporting data for experiments with optogenetic stimulation of glutamatergic PPN neurons and concomitant chemogenetic inhibition of glutamatergic CnF neurons.

(a) Mapping of injection spread. For each mouse (n = 10) the infection site was identified and segmented as a series of coordinates forming contour maps for sequential rostrocaudal bregma level. Maps were superimposed with overlapping areas showing a darker shade. Mice were injected with 2 viruses: ChR2 targeting the caudal Pedunculopontine nucleus (PPN, red), and inhibitory DREADD ('iD') targeting the Cuneiform nucleus (CnF, purple). Scale 1mm.

(b) Anatomical fiber localization for the cohort presented in **a**. Distance from Bregma indicated under each scheme (scale 1mm). Fiber ends are color coded representing each mouse (M1-10).

(c) Testing effect of CnF inhibition in the Open Field. Bar graph with before-after lines shows total distance moved (Saline, grey vs CNO, purple) (two-tailed, paired t-test). Line graph shows distance moved over time (mean ± SEM, Two-way RM ANOVA, Geisser Greenhouse correction followed by Bonferroni's multiple comparison, report shows post hoc results).

(d) Percentage of locomotor time (>2cm/s) in the Open Field (two-tailed, paired t-test). Data are presented as mean ± SD.

(e) Open Field. Percentage of time on each speed range. Heat map with individual mice in horizontal lines. No differences observed between Saline and CNO conditions (Two-way RM ANOVA, Geisser Greenhouse correction followed by Bonferroni's multiple comparison, report shows overall post hoc results).

(f-j) Experiments with concomitant CNO and optogenetics.

(f) Control saline (left), haloperidol (center) and SCH (right) experiments. Min-to-max boxplots show latency (s) to initiate locomotion. Individual points indicate average value per animal during baseline and injected period (Wilcoxon matched pairs signed rank test, two-tailed).

(g) Control saline (left), haloperidol (center) and SCH (right) experiments. Connected lines show the fraction of trials where stimulation initiated a locomotor bout before and after injection (two-tailed, paired t-test).

(h) Timeline for optogenetic experiment highlighting non-stimulus periods within the Open Field protocol used to confirm parkinsonian state development: Habituation (Hab ①) and latency to session end (Latency ②). Note prolonged (20min) waiting point to allow for CNO effect. Immediately after the Open Field session, mice performed the Bar test. Lower panel shows in sequence: saline, haloperidol and SCH injected sessions (mean ± SEM). Distance travelled was normalized to Hab average (Wilcoxon matched-pairs, one-tailed).

(i) Bar test. Graph shows impaired capacity to initiate movement (time limit, 20s) in mice treated with CNO followed by halo or SCH (mean ± SEM). Individual values represent average of 3 sequential trials per mouse. If aided by the laser, mice could descend the bar (Wilcoxon matched-pairs, one-tailed).

(j) Normalized distance travelled (%) of 'pre', 'laser' and 'post' epochs in the Wavelength dependency test (593nm). Grey lines represent the average of eight trials for each mouse and group mean ± SEM in purple (Friedman test -RM, followed by Dunn's comparison to 'pre', with graph report of multiple comparison).

Data composed of 10 Vglut2^{cre} mice injected with inhibitory DREADDs in the CnF (bilateral) and ChR2 in the PPN (unilateral). halo: haloperidol [D2-antagonist]; SCH: SCH23390 [D1-antagonist]. See also **Fig.7** and detailed stats in **Table S3**. Source data are provided as a Source Data file.

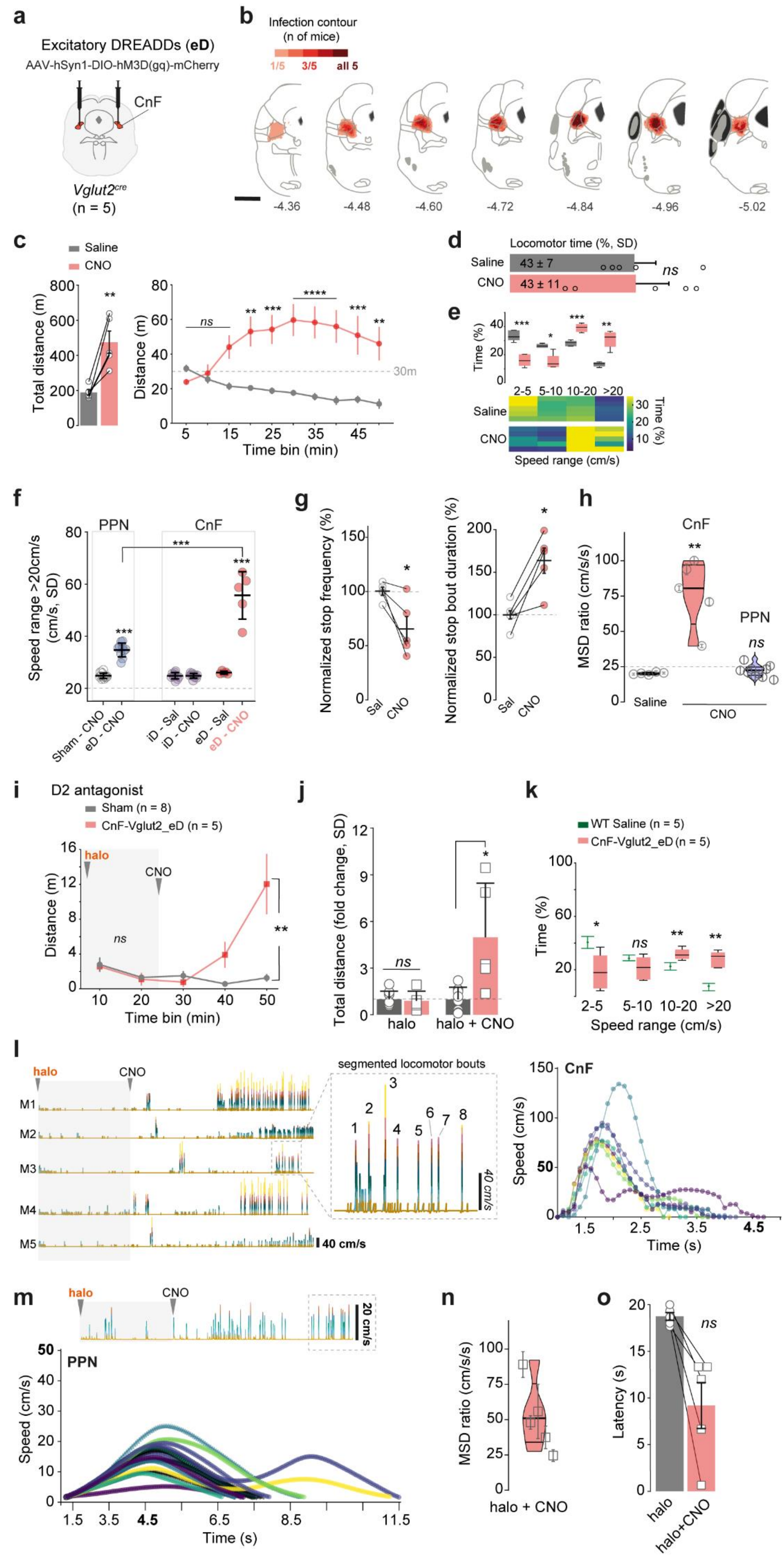
a
Excitatory DREADDs (eD)
AAV-hSyn1-DIO-hM3D(gq)-mCherry
CnF
Vglut2cre
(n = 5)
b
Infection contour
(n of mice)
1/5
3/5
all 5
-4.36
-4.48
-4.60
-4.72
-4.84
-4.96
-5.02
c
Saline
CNO
Total distance (m)
Distance (m)
Time bin (min)
30m
d
Locomotor time (%, SD)
Saline
CNO
43 ± 7
43 ± 11
ns
e
Time (%)
2-5
5-10
10-20
>20
Speed range (cm/s)
f
PPN
CnF
Speed range >20cm/s (cm/s, SD)
Sham - CNO
eD - CNO
iD - Sal
iD - CNO
eD - Sal
eD - CNO
g
Normalized stop frequency (%)
Normalized stop bout duration (%)
Sal
CNO
h
CnF
PPN
MSD ratio (cm/s/s)
Saline
CNO
i
D2 antagonist
Sham (n = 8)
CnF-Vglut2_eD (n = 5)
halo
CNO
Distance (m)
Time bin (min)
j
Total distance (fold change, SD)
halo
halo + CNO
k
WT Saline (n = 5)
CnF-Vglut2_eD (n = 5)
Time (%)
Speed range (cm/s)
l
halo
CNO
M1
M2
M3
M4
M5
40 cm/s
segmented locomotor bouts
CnF
Speed (cm/s)
Time (s)
m
halo
CNO
20 cm/s
PPN
Speed (cm/s)
Time (s)
n
MSD ratio (cm/s/s)
halo + CNO
o
Latency (s)
halo
halo+CNO

Supplementary Fig. 6 (S6)│ Data from chemogenetic experiments targeting glutamatergic Cuneiform (CnF) neurons.

(a) Viral strategy to bilaterally express excitatory DREADDs (‘eD’) in Vglut2-CnF neurons (n = 5 mice).

(b) Mapping of injection spread. For each mouse the infection site was identified and segmented as a series of coordinates forming contour maps for each rostrocaudal bregma level. Maps were superimposed with overlapping areas showing a darker shade. Scale 1mm.

(c) Bar graph with before-after lines shows total distance moved in the Open Field test for saline (grey) and CNO (red) injected mice (two-tailed, paired t-test). Line graphs shows timeline effect of CNO (Two-way RM ANOVA, Geisser Greenhouse correction followed by Bonferroni’s multiple comparison, report shows post hoc results). Data are presented as mean ± SEM.

(d) Percentage of locomotor time in saline (grey) and CNO (red) treated mice (>2cm/s) (two-tailed, paired t-test). Data are presented as mean ± SD.

(e) Upper panel shows speed range in saline (grey) and CNO (red) injected mice with min-to-max boxplots (Two-way RM ANOVA, Geisser-Greenhouse correction, with report for Bonferroni’s multiple comparison). Lower panel shows heat map with individual mice in horizontal lines and color code for percentage of time in each speed range.

(f) For all experiments with chemogenetics mice in the Open Field, we calculated the average speed used within the highest speed range (i.e., whenever above 20cm/s) (Kruskal-Wallis with report for Dunn’s multiple comparisons to Sham group. Direct comparison between PPN and CnF performed with Mann-Whitney). For group numbers see end of legend.

(g) Stop events in Vglut2^{cre} mice injected with eD in CnF counted across the 50min Open Field. Data was normalized to saline average (dashed line). Individual data points (circles) and group mean ± SEM (line) are shown. Left panel, stop frequency (35.27% decrease upon CNO). Right panel, average stop bout duration (67.20% increase, Two-tailed, paired t-test).

(h) MSD ratios in Vglut2^{cre} mice injected with eD in CnF (n = 5) or PPN (n = 10). Dashed line marks ratio found amongst WT mice by other labs[50]. The violin plots show the median and quartiles of this dataset for Vglut2-CnF targeted (+CNO in red; saline injected in grey) and Vglut2-PPN targeted (+CNO, blue) mice. Individual mice (circles) are plotted together with confidence intervals (CI 95%). Kruskal-Wallis with graph reporting for Dunn’s multiple comparisons.

(i) Timeline effect of D2 antagonist, haloperidol (‘halo’) before (light-grey background) and after injection of CNO in Sham and eD Vglut2-CnF mice (Two-way RM ANOVA with graph reporting group effect and analysis done for each period separately). Data are presented as mean ± SEM.

(j) Fold differences in total distance travelled in the Open Field. Data was normalized to Sham (grey) and eD Vglut2-CnF (red) mice after haloperidol injection and upon CNO treatment (two-tailed, t-test with Welch’s correction, analysis done for each period separately).

(k) Percentage of time in each speed range for Vglut2-CnF targeted (red) mice after halo and CNO treatment in comparison to WT saline group (green). Min-to-max boxplots (Two-way RM ANOVA, Geisser-Greenhouse correction, report shows Sidak’s vs WT$^{sal+sal}$).

(l) Left panel shows the instantaneous speed profile for all tracks of mice presented in **panel i** (speed follows divergent color scheme to facilitate reading). Mice were injected with halo followed by CNO and show abrupt changes to high speed locomotion. Center panel shows magnification for mouse 3 (M3) and segmentation of 8 occurrences of locomotor bout, which are then plotted in the right panel showing the speed (cm/s) as a function of time from start to end of each bout.

(m) Upper panel shows track from Vglut2^{cre} mouse injected with eD targeting the PPN (data from **Fig.2b**). Dashed square shows area from which example locomotor bouts were extracted to plot speed profile from start to end of each bout in the lower panel. Note the difference in duration and maximal values reached in comparison to **panel i** (sampling rate, circles, is equal between graphs).

(n) MSD ratio of *Vglut2^{cre}* mice injected with eD in CnF and given haloperidol followed by CNO. Individual mice (grey squares) are plotted together with confidence intervals (95% CI). The violin plot shows the median and quartiles of this dataset.

(o) Bar test. Graph shows impaired capacity to initiate movement (time limit, 20s) in mice with chemogenetic activation of CnF. Individual values represent average of 3 sequential trials per mouse. Upon CNO injection an overall decrease in latency occurs

(median difference with decrease of 6.7s after CNO), yet individual responses show high variability on repeated testing (coefficient of variation: halo only 5.095%, upon CNO, 59.84%). As a result, no significant difference is found (two-tailed, Wilcoxon matched-pairs, $p=0.0625$, $W= -15.00$).

Data composed of 5 Vglut2^{cre} mice injected with eD in the CnF and treated with saline or CNO. Extra groups extracted to serve as references from previous experiments included 5 WT$^{sal+sal}$; 8 Sham$^{halo+CNO}$; 10 Vglut2_eD^{CNO} (PPN) and 10 Vglut2^{cre} mice injected with iD in the CnF and treated with saline or CNO. See detailed stats in **Table S4**. Source data are provided as a Source Data file.

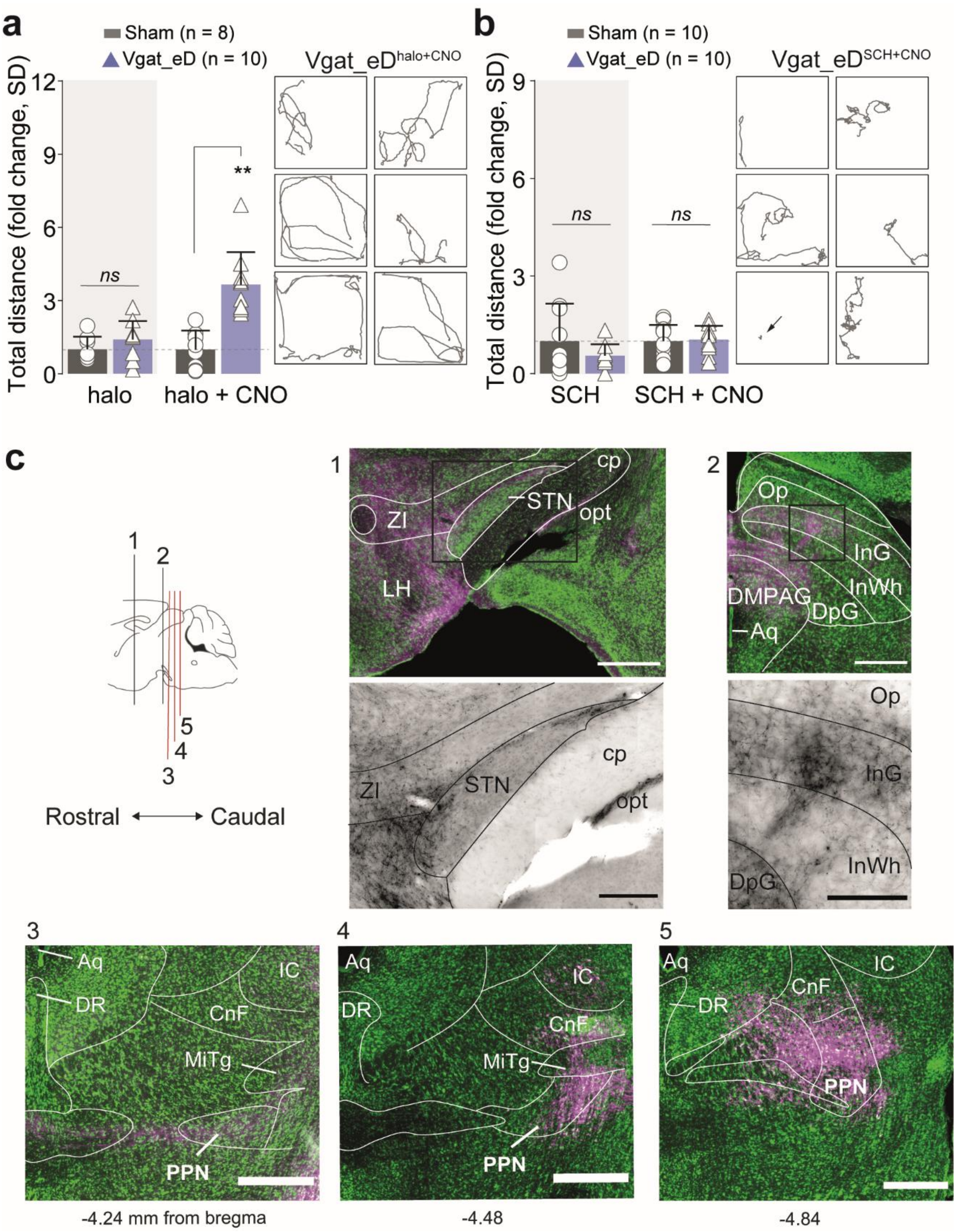

Supplementary Fig. 7 (S7)│ Supporting data for experiments with Vgat_eD mice showing cumulative data in the Open Field, anatomical projections and caudal targeting.

(a) Left panel shows fold differences in total distance travelled in the Open Field. Data was normalized to Sham (grey) and Vgat_eD (blue) mice after D2 receptor antagonism with haloperidol ('halo') and upon CNO treatment (two-tailed, t-test with Welch's correction, analysis done for each period separately). Right panel shows traces for CNO treated Vgat_eD mice during the last 5 min of recording (minute 45 to 50). Six mice per group were randomly selected as examples.

(b) Left panel shows fold differences in total distance travelled in the Open Field. Data was normalized to Sham (grey) and Vgat_eD (blue) mice after D1 receptor antagonism with SCH23390 ('SCH') and upon CNO treatment (two-tailed, t-test with Welch's correction, analysis done for each period separately). Right panel shows traces for CNO treated Vgat_eD mice during the last 5 min of recording (minute 45 to 50). Six mice per group were randomly selected as examples.

(c) Microscope images of five representative coronal slices with neurons in green and DREADD-expressing $Vgat^{+}$ cells stained in magenta. Upper panels show ascending (1-2) fiber projections of GABAergic PPN neurons. Lower panels (3-5) show rostral to caudal cell distribution with high neuronal concentration in the caudal part. High magnification insets (black squares in panels '1-2') show fibers within STN (highest density in medial part including parasubthalamic area) and InG (here only magenta channel is shown, inverted LUT). Scale 500µm, inset 250µm. Boundaries based on Mouse brain atlas[117].

Data are presented as mean ± SD. 8 $Sham^{halo+CNO}$; 10 $Sham^{SCH+CNO}$; 10 $Vgat\_eD^{halo+CNO}$; 10 $Vgat\_eD^{SCH+CNO}$ mice. **(a, b)** Two-way RM ANOVA, followed by Bonferroni's multiple comparison vs Sham, analysis done for each period separately. See also **Fig.9** and detailed stats on **Table S5**. **c(1)** ZI: zona incerta -ventral part, LH: lateral hypothalamic area, cp: cerebral peduncle, opt: optic track. **c(2)** Superior colliculus - Op: Optic nerve layer, InG: intermediate grey layer, InWh: intermediate white layer, DpG: deep grey layer, DMPAG; dorsomedial periaqueductal grey, Aq: aqueduct. **c(3-5)** DR: dorsal raphe, IC: inferior colliculus, CnF: cuneiform nucleus and precuneiform nucleus, MiTg: microcellular tegmental nucleus, PPN: pedunculopontine nucleus. Source data are provided as a Source Data file.

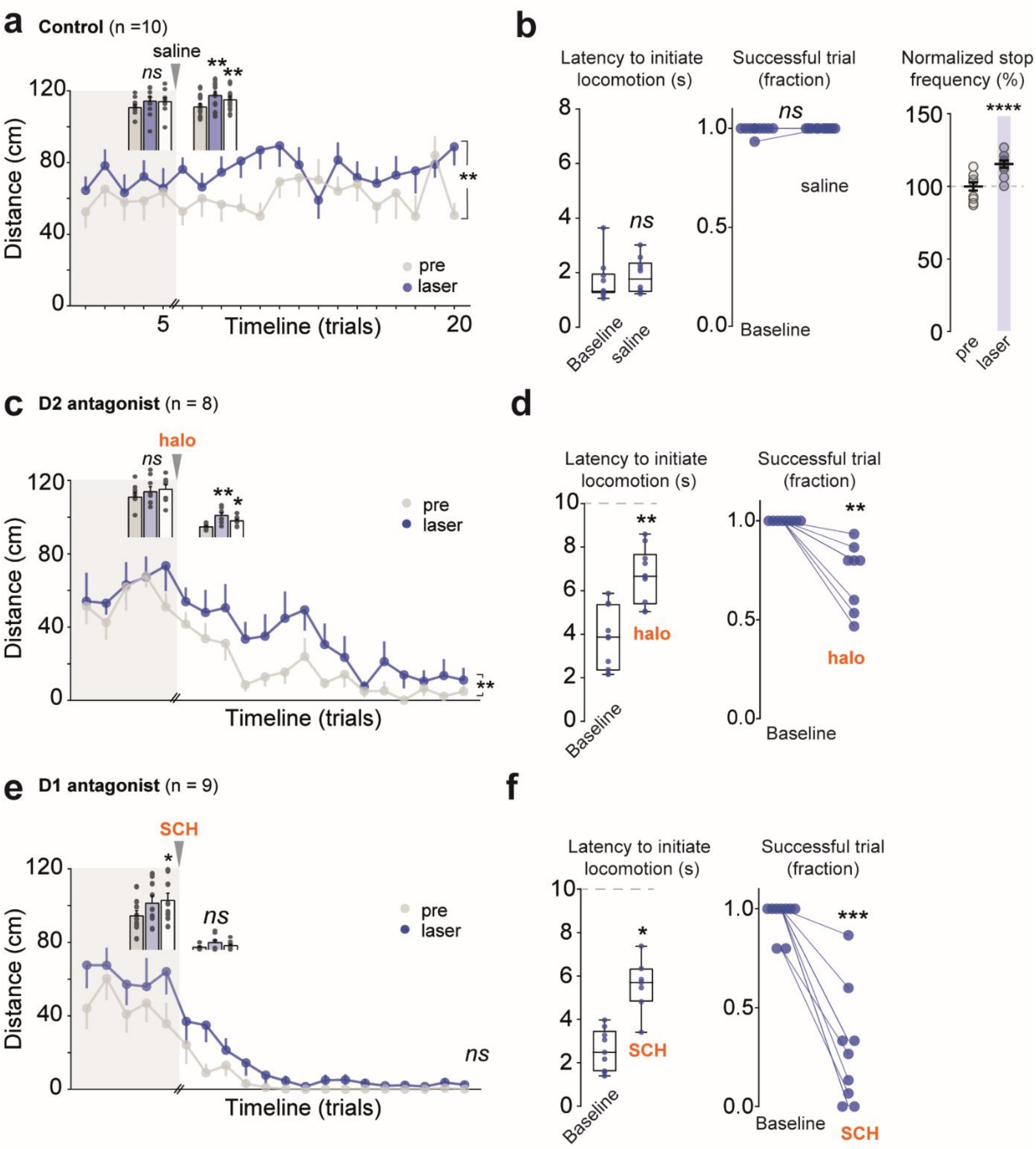

Supplementary Fig. 8 (S8)│ Optogenetic experiments with Vgat_ChR2 mice showing Parkinsonian-like motor impairments induced by dopamine receptor antagonists.

(a) Control experiment with saline injection in Vgat_ChR2 mice. Line graph shows distance moved in each trial throughout baseline (light-grey background) and after saline injection, for 'pre' (grey) and 'laser (blue) epochs. Inset bars show the average distance travelled during each epoch (pre, laser, post) for both periods. Same scale applies to both bar sets. Data presented as group mean ± SEM.

(b) Saline-injected Vgat_ChR2 mice. Left panel, min-to-max boxplot shows latency (s) to initiate locomotion. Individual points represent average value per animal during baseline and saline period (Wilcoxon matched pairs signed rank test, two-tailed). Center panel shows the fraction of trials where locomotor bouts were detected before and after saline (paired t-test, two-tailed). Right panel shows stop frequency during 'laser' epoch, normalized to 'pre' ($p<0.0001$, two-tailed, paired t-test, $t=7.889$, $df=9$ with pairing $r = 0.7513$, mean ± SEM and individual values).

(c) D2 antagonist haloperidol (‘halo’) experiment. Line graph shows distance moved during ‘pre’ and ‘laser’ epochs, throughout baseline (light-grey background) and PD challenge trials. Inset bars show the average distance travelled per epoch in each period (same scale for both sets). Data presented as group mean ± SEM.

(d) Latency (s) to initiate locomotion during opto-stimulation (left, min-to-max boxplot) and fraction of opto-stimulation trials where locomotor bouts were detected (right) in haloperidol injected Vgat_ChR2 mice. Stats and parameters same as in **b**.

(e) D1 antagonist SCH23390 (‘SCH’) experiment. Line graph shows distance moved during ‘pre’ and ‘laser’ epochs through baseline (light-grey background) and PD challenge trials. Inset bars show the average distance travelled per epoch in each period (same scale for both sets). Data presented as group mean ± SEM.

(f) Latency (s) to initiate locomotion during opto-stimulation (left, min-to-max boxplot) and fraction of opto-stimulation trials where locomotor bouts were detected (right) in SCH injected Vgat_ChR2 mice. Stats and parameters same as in **b**.

Statistics (a, c, f) Line graphs: Two-way RM ANOVA (laser condition vs trial timeline, only events 6 to 20), report shows the main effect of laser condition. **Insets:** Bar graph analysis is done on all six conditions concomitantly as Two-way RM ANOVA, Geisser-Greenhouse correction, report shows Dunnett’s multiple comparisons to ‘pre’. Protocol is the same as **Fig.6**. See also **Movie 2**, **Fig.S9** and detailed stats in **Table S6**. Source data are provided as a Source Data file.

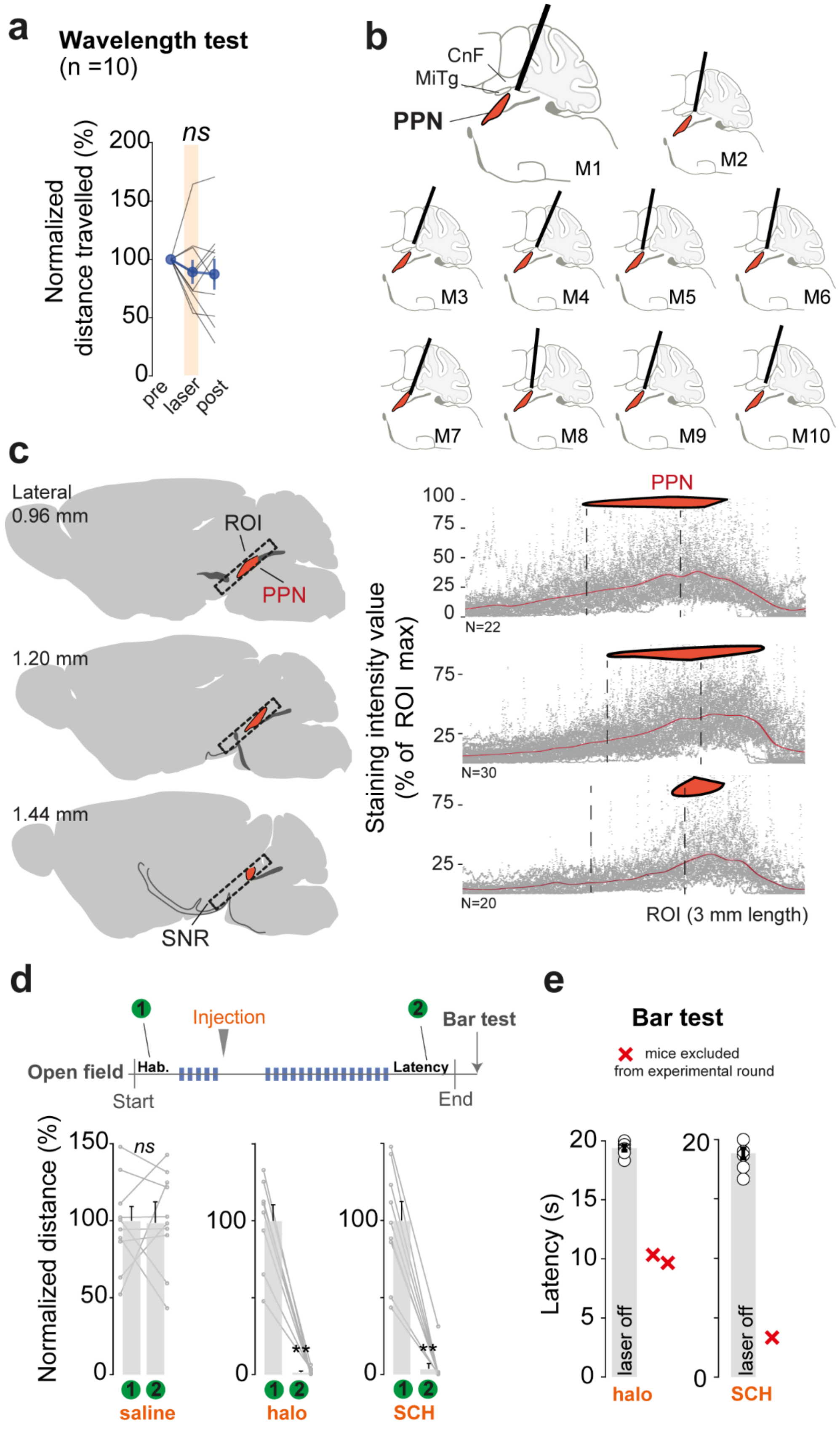
a
Wavelength test
(n =10)
ns
Normalized distance travelled (%)
200
150
100
50
0
pre
laser
post
b
CnF
MiTg
PPN
M1
M2
M3
M4
M5
M6
M7
M8
M9
M10
c
Lateral
0.96 mm
ROI
PPN
1.20 mm
1.44 mm
SNR
PPN
Staining intensity value (% of ROI max)
100
75
50
25
0
N=22
N=30
N=20
ROI (3 mm length)
d
Injection
Bar test
Open field
Hab.
Latency
Start
End
Normalized distance (%)
ns
**
saline
halo
SCH
e
Bar test
mice excluded from experimental round
Latency (s)
laser off
halo
SCH

Supplementary Fig. 9 (S9)│ PPN injected Vgat_ChR2 mice. Wavelength dependency test, anatomical reports and variables confirming the akinetic phenotype during dopamine signaling depletion.

(a) Normalized distance travelled (%) of 'pre', 'laser' and 'post' epochs in the Vgat_ChR2 wavelength dependency test (593nm). Grey lines represent the average of eight trials for each mouse and the blue line represents group mean (Friedman test -repeated measures, followed by Dunn's comparison to 'pre', with graph report of multiple comparison).

(b) Anatomical fiber localization for the Vgat_ChR2 mice included in the study. Sagittal mediolateral position = 1.20mm.

(c) Rostro to caudal quantification of staining intensity in Vgat_ChR2 cohort shown in **b**. Three Sagittal segments were selected for analysis (0.96, 1.2 and 1.44mm lateral from bregma). Using a rectangular region of interest (ROI, 3mm length, 500µm height) placed over the PPN, we extracted the staining intensity values for 2-3 sections/segment/mouse (e.g., lateral 1.20mm = 3sections/mouse, in 10 mice N=30 slices in total).

(d) Timeline for optogenetic experiment highlighting the non-stimulus periods within the Open Field protocol used to confirm parkinsonian state development: Habituation ('Hab' ①) and latency to session end ('Latency' ②). Immediately after the Open Field session, mice performed the Bar test. Lower panel shows in sequence: saline (left), haloperidol (center) and SCH (right) injected experimental sessions. Distance travelled was normalized to habituation average (Wilcoxon matched-pairs, one-tailed, saline p=0.3477, halo p=0.0039, SCH p=0.0020).

(e) Bar graph shows impaired capacity to initiate movement in Vgat_ChR2 (time limit, 20s) mice challenged with halo or SCH. Individual values represent average of 3 sequential trials per mouse. Red crosses indicate the average latency in mice that were excluded from the data set for not showing sufficiently robust akinetic phenotype in order to access recovery (see criteria on methods).

Data are presented as mean ± SEM. Saline experiment = 10 mice; Halo = 8; SCH = 9. halo: haloperidol [D2-antagonist]; SCH: SCH23390 [D1-antagonist]. In **b**, CnF: cuneiform nucleus and precuneiform nucleus, MiTg: microcellular tegmental nucleus, PPN: pedunculopontine nucleus. See also **Movie 2**, **Fig.S8** and detailed stats in **Table S6**. Source data are provided as a Source Data file.

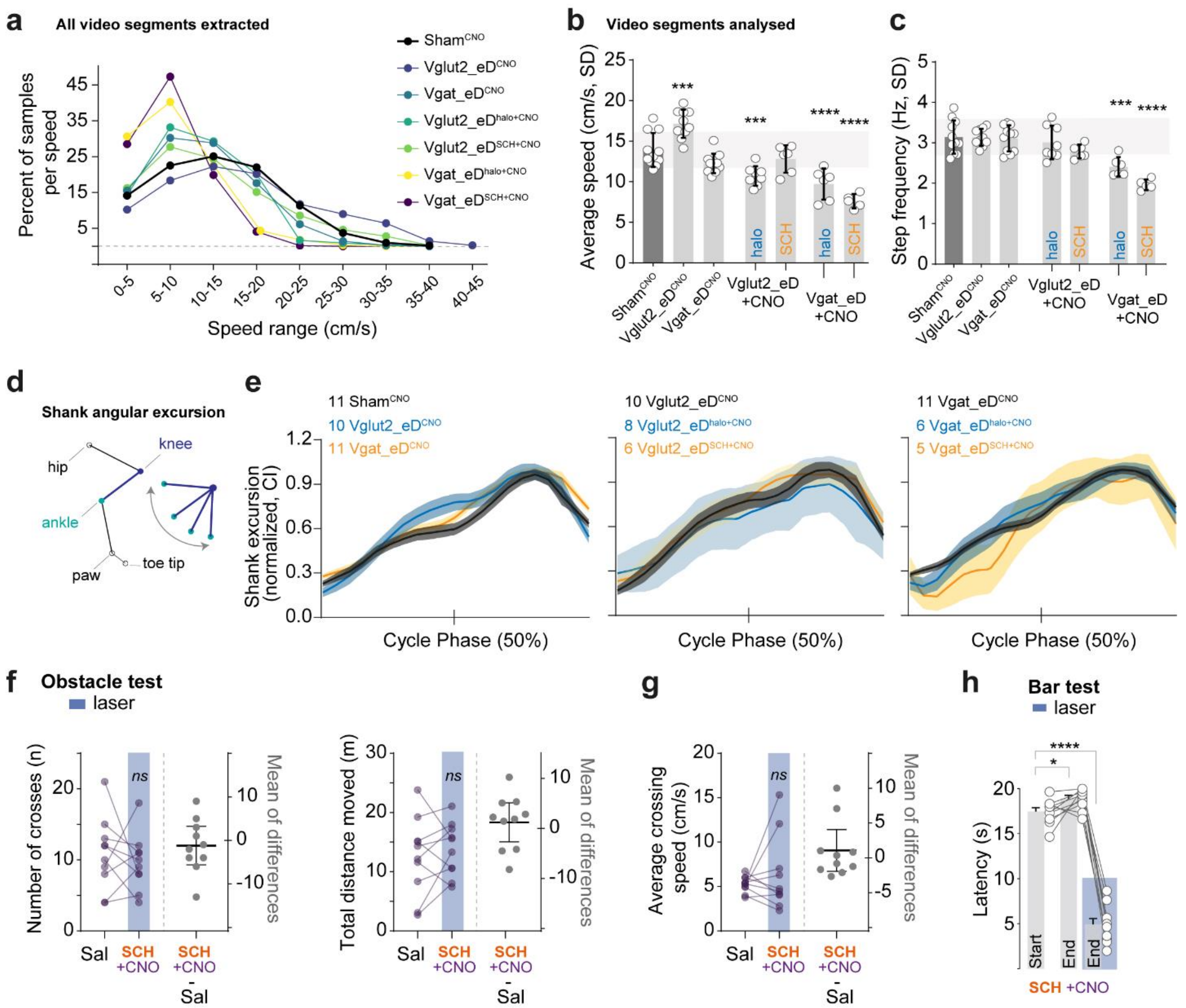

Supplementary Fig. 10 (S10) | Supporting data for motor proficiency tests.

(a) Average speed ranges of each experimental group used for Kinematic analysis. Data is based on all extracted video segments from the Open Field chemogenetic experiments. Only a sub-set was used for final analysis.

(b) Individual average speed based on samples used for intralimb Kinematic analysis. Horizontal shadow bar indicates data range for Sham^{CNO} (One-way RM ANOVA, followed by Dunnett's multiple comparison to Sham^{CNO} in dark grey, report shows post hoc results).

(c) Step frequency (Hz) from data in **b**. Calculation based on touchdown events (hind paw initiating contact with floor). Horizontal shadow bar indicates data range for Sham^{CNO} (One-way RM ANOVA, followed by report of Dunnett's multiple comparison to Sham^{CNO} in dark grey).

(d) Stick diagram showing all joints tracked in one hindlimb. The Shank is represented by the vector connecting knee to ankle.

(e) Normalized angular excursion of the Shank plotted as a function of step cycle. Left panels shows control condition (CNO-only). Groups mean (line) and confidence interval (95%, shadow zones).

(f) Obstacle test, control variables. Left panel (before-after purple lines) show number of corridor crosses performed by each subject under saline or challenge (blue) condition. In grey, we computed the difference between pairs (one circle per mouse, mean ± CI, in black value is close to zero). Right panel, total distance covered. Stats report for two-tailed, paired t-test: number of crosses p=0.5543, t=0.6142, df=9; distance covered p=0.5015, t=0.7002, df=9.

(g) Average speed during full corridor crosses in the Obstacle test (mean ± CI, in black, two-tailed, paired t-test, p=0.4390, t=0.8096, df=9).

(h) Bar test results in mice before and after performing the Obstacle test challenge. Bar graph shows impaired capacity to initiate movement in mice injected with SCH and CNO (i.e., confirming akinetic state prior recordings in the Obstacle test start). Immediately after, mice were tested for akinesia again, to confirm that akinesia was present through the recording period. Finally, the laser was activated (+laser) and mice were capable to descend the bar (Two-way RM ANOVA with report for Bonferroni). Symbols represent average of 3 sequential trials per mouse, bars show group mean ± SEM.

Data composed of (a-e) Mice from experiments reported in **Fig.1-3** and **9**, number of mice reported within panel **e**; **(f-h)** same mice cohort as in **Fig.7**, n = 10 mice. halo: haloperidol [D2-antagonist]; SCH: SCH23390 [D1-antagonist]. See also **Fig.10** and detailed stats in **Table S7**. Source data are provided as a Source Data file.

Supplementary Table 1. Statistical details of Vglut_eD experiments.

Group (n)	Figure/Result Claim	Outcome measured	Statistical approach	Descriptive report	Exact P and critical values
Vglut2_eD^{CNO} =5 [unilateral], Vgat_eD^{CNO} =5 [unilateral], WT^{CNO} =2	**Fig.S1d (c-Fos)**	Mean count/per mouse/hemisphere	Two-way RM ANOVA, matched by mouse and comparing hemispheres, Sidak's	ANOVA hemisphere effect $F_{(1, 9)}$ = 31.35, p=0.0003	Sidak's ipsi vs contra: [Vglut2_eD] p=0.0002, [Vgat_eD] p=0.0032, [WT] p>0.9999
			Brown-Forsythe and Welch ANOVA, Dunnett (T3) comparing contralateral hemisphere (vs WT)	Difference between means: WT/Vglut = -61.25 ± 17.28 (SE); CI = -133.7 to 11.24 and WT/Vgat = -16.43 ± 3.265 (SE); CI = -27.77 to -5.086	Brown-Forsythe ANOVA = $9.459_{(2.000, 4.283)}$ with p=0.0268; Welch's ANOVA = $16.56_{(2.000, 6.289)}$ with p=0.0031; Dunnett Vglut p=0.0432, Vgat p=0.0045
	**Fig.S1e (c-Fos)**	Colocalization/per mouse (%) [injected hemisphere-only]	two-tailed, paired t-test [Vglut2_eD]	Colocalized, MEAN± SEM / Median = 76.59±3.758 / 75.61%	p=0.0002, t=7.076, df=4
			two-tailed, paired t-test [Vgat_eD]	Colocalized, MEAN± SEM / Median = 84.76±2.163 / 84.62%	p<0.0001, t=16.07, df=4
	**Fig.S1f (c-Fos)**	Total distance moved (m) after CNO injection [bins 35-85min]	Brown-Forsythe and Welch ANOVA, Dunnett (T3) (vs WT)	Difference between means: WT/Vglut = --217.8 ± 38.54 (SE); CI = -379.5 to -56.11 and WT/Vgat = -61.68 ± 4.009 (SE); CI = -78.50 to -44.86	Brown-Forsythe ANOVA = $21.35_{(2.000, 4.085)}$ with p=0.0069; Welch's ANOVA = $119.6_{(2.000, 5.489)}$ with p<0.0001; Dunnett Vglut p=0.0088, Vgat p=0.0002
Sham^{CNO} =15, WT^{Sal} =5	**CNO dose has no effect in Sham mice**	Total distance moved (m)	two-tailed, unpaired t-test with Welch's correction	MEAN± SEM> Sham = 129.2 ± 9.805; WT = 167.0 ± 19.98	p=0.1397; t=1.699, df=6.059
		Distance moved (m) per time bin	Two-way RM ANOVA, Geisser Greenhouse correction, Bonferroni		Group x Time $F_{(9, 162)}$ = 1.131 with p=0.3435
Vglut2_eD^{CNO} =10	**Fig.1b, peak at bin 15 (20min after CNO)**	Distance moved (m) per time bin	One-way ANOVA, Friedman test		Bin 5 vs 15, p=0.0221; Bin 10 vs 15, p=0.0461; no effect thereafter
	**Fig.1b, maintained through 50min session**		One-way RM ANOVA, Geisser Greenhouse correction, test for trend (time bin)	Linear trend $F_{(1, 81)}$ = 2.028 with p=0.1583. Slope = -0.2553; SE of slope = -0.1793; CI of slope = 0.1014 to -0.6119 with p=0.1583	ANOVA Time effect: $F_{(3.044, 27.39)}$ = 1.881 with p=0.1556
Sham^{CNO} =15, Vglut2_eD^{CNO} =10	**Fig.1b, left panel**	Total distance moved (m)	two-tailed, unpaired t-test	MEAN ± SEM > Vglut = 245.7 ± 21.29 and Difference between means = 116.5 ± 21.02 (SEM); CI = 73.05 to 160.0	p<0.0001; t=5.544, df=23

Group (n)	Figure/Result Claim	Outcome measured	Statistical approach	Descriptive report	Exact P and critical values
	**Fig.1b, right panel**	Distance moved (m) per time bin	Two-way RM ANOVA, Geisser Greenhouse correction, Bonferroni	ANOVA group effect $F_{(1, 23)}$ = 30.73	Group x Time $F_{(9, 207)}$ = 5.249 with p<0.0001
	**Fig.1c**	Time locomoting (%) (>2cm/s)	two-tailed, unpaired t-test	Difference between means = 11.09 ± 3.326 (SEM); CI = 4.213 to 17.97	p=0.0029; t=3.335, df=23
	**Fig.1d**	Percentage of time in each speed range when locomoting (%)	Two-way RM ANOVA, Geisser-Greenhouse correction, Bonferroni	Difference between means/speed range; [2-5]= 18.04, CI =12.63 to 23.45, [5-10]= 8.638, CI = 4.421 to 12.85, [10-20]=-10.73, CI = -13.90 to -7.552, [>20]=-15.95, CI = -22.28 to -9.627	Group x speed range $F_{(3, 69)}$ = 79.79 with p<0.0001
$Sham^{halo+CNO}$ =8, Vglut2_$eD^{Halo+CNO}$ =10	**Fig.2c**	Distance moved (m) per time bin [before CNO]	Two-way RM ANOVA		Group effect $F_{(1, 16)}$ = 0.7229 with p=0.4077
		Distance moved (m) per time bin [after CNO]	Two-way RM ANOVA		Group effect $F_{(1, 16)}$ = 7.895 with p=0.0126
	**Fig.S1g**	Fold change of total distance moved per experimental period	two-tailed, unpaired t-test, Welch's correction	MEAN ± SEM> [Halo only] Sham = 1 ± 0.1830; Vglut = 1.282 ± 0.2570; [after CNO] Sham = 1 ± 0.2731; Vglut = 3.582 ± 0.7880	[Halo only]p=0.3853; t=0.8937, df=15.36 [after CNO] p=0.0050; t=3.096, df=11.09
$Sham^{halo+CNO}$ =8, Vglut2_$eD^{halo+CNO}$ =10, $Wt^{sal+sal}$ =5	**Fig.2d**	Percentage of time in each speed range when locomoting (%)	Two-way RM ANOVA, Geisser-Greenhouse correction, Dunnett (vs $WT^{sal+sal}$)	Difference between means [speed range] WTxSham/WTxVglut; [2-5]= -35.39/0.1996, [5-10]= 13.79/5.545, [10-20]= 15.79/-1.401, [>20]= 5.814/-4.343	Group x speed range $F_{(6, 60)}$ = 10.65 with p<0.0001
$Sham^{halo+CNO}$ =8, Vglut2_$eD^{halo+CNO}$ =10	**Fig.2e**	Latency (s) to descent on bar test	two-tailed, non-matched, Mann Whitney test	MEDIAN > Sham = 18.67; Vglut = 1.167. Median difference (Hodges-Lehmann) = -17.67	p<0.0001 [calculated with ties among values]
$Sham^{halo+CNO}$ =8		Latency (s) to descent on bar test	One sample Wilcoxon test, theoretical median 20s	MEAN ± SEM > Sham = 18.79 ± 0.2668	Discrepancy = 0, CI = -2.000 to 0.000 with actual confidence level = 97.73%; p=0.0020
$Sham^{SCH}$ =10, Vglut2_eD^{SCH} =10	**Fig.3a**	Distance moved (m) per time bin [before CNO]	Two-way RM ANOVA		Group effect $F_{(1, 18)}$ = 3.003 with p=0.1002
		Distance moved (m) per time bin [after CNO]	Two-way RM ANOVA		Group effect $F_{(1, 18)}$ = 10.93 with p=0.0039
	**Fig.S1h**	Fold change of total distance moved per experimental period	two-tailed, unpaired t-test, Welch's correction	MEAN ± SEM> [SCH only] Sham = 1 ± 0.3648; Vglut = 1.917 ± 0.3836; [after CNO] Sham = 1 ± 0.1583; Vglut = 2.354 ± 0.3375	[SCH only]p=0.1003; t=1.733, df=17.95 [after CNO] p=0.0031; t=3.307, df=12.07
$Sham^{SCH+CNO}$ =10, Vglut2_$eD^{SCH+CNO}$ =10, $Wt^{sal+sal}$ =5	**Fig.3b**	Percentage of time in each speed range when locomoting (%)	Two-way RM ANOVA, Geisser-Greenhouse correction, Dunnett (vs $WT^{sal+sal}$)	Difference between means [speed range] WTxSham/WTxVglut; [2-5]= -27.10/14.10, [5-10]= 9.289/7.320, [10-20]= 12.37/-8.764, [>20]= 5.442/-12.65	Group x speed range $F_{(6, 66)}$ = 15.30 with p<0.0001

Group (n)	Figure/Result Claim	Outcome measured	Statistical approach	Descriptive report	Exact P and critical values
$Sham^{SCH}$ =10, $Vglut2\_eD^{SCH}$ =10	**Fig.3c**	Latency (s) to descent on bar test	two-tailed, non-matched, Mann Whitney test	MEDIAN > Sham = 18.67; Vglut = 1.00. Median difference (Hodges-Lehmann) = -17.00	p<0.0001 [calculated with ties among values]

Supplementary Table 2. Statistical details of Calcium Imaging experiments. Striatum (STR), Pedunculopontine nucleus (PPN).

Group (n)	Figure/Result Claim	Outcome measured	Statistical approach	Descriptive report	Exact P and critical values
Drd1_STR =4, Vglut2_PPN =4	**Decline in neuronal calcium fluctuation after drug injection**	Z-score (units of SD) decline over 50s bin [starts 150s prior inj.]	Spearman's Rank-Order correlation	95% CI > STR^{halo} -0.9798 to -0.9147, STR^{SCH} -0.9778 to -0.9067, PPN^{halo} -0.9380 to -0.7549, PPN^{SCH} -0.9819 to -0.9234	STR^{halo} r=-0.9582, STR^{SCH} r=-0.9542, PPN^{halo} r=-0.8747, PPN^{SCH} r=-0.9626 all with p<0.0001
Drd1_STR =4	**Fig.4d**	Distance moved (cm) per time bin (3min/each)	Two-way RM ANOVA, matched by test day and time bin, Sidak's	Interaction $F_{(13, 39)}$ = 0.7449 with p=0.7092, Sidak's ns for all bin comparisons	Time bin $F_{(13, 39)}$ = 47.42 with p<0.0001 accounts for 87.46% of total variance
Vglut2_PPN =4	**Fig.S2c**			Interaction $F_{(13, 39)}$ = 0.9368 with p=0.5263, Sidak's ns for all bin comparisons	Time bin $F_{(13, 39)}$ = 17.07 with p<0.0001 accounts for 74.13% of total variance
Drd1_STR =4, Vglut2_PPN =4	**Fig.S2d**	Individual mice, average Z-score (units of SD)	Two-way RM ANOVA, matched by test period and drug, Fisher's LSD	Difference between means > drug-free x PD; STR 0.5424, CI = 0.3421 to 0.7428. PPN 0.6083, CI = 0.3445 to 0.8721	Interaction STR $F_{(1, 3)}$ = 0.03305 with p=0.8673, PPN F (1, 3) = 33.20 with p=0.0104
Drd1_STR =4	**Fig.S4f [STR related]**	Cells classified by response type and their average Z-score compared during drug-free and PD periods	Two-way ANOVA, Tukey's comparing all conditions	Interaction $F_{(3, 640)}$ = 285.8 with p<0.0001 corresponding to 34.5% of the total variation. Response type $F_{(1, 640)}$ = 7.028 with p=0.0082 (0.2828% of total variation). Test period vs Cell type & genotype $F_{(3, 640)}$ = 19.16 with p<0.0001 (2.313% of total variation)	Tukey's response type classification: Decrease or Increase type, all p<0.0001 (these p values show correct classification approach). Tukey's comparing Z-score between response types (referred to colors in graph); [Halo] grey vs green p<0.0001, green vs grey p=0.2329. [SCH] grey vs green p<0.0001, green vs grey p=0.1970
Vglut2_PPN =4	**Fig.S4f [PPN related]**	Cells classified by response type and their average Z-score compared during drug-free and PD periods	Two-way ANOVA, Tukey's comparing all conditions	Interaction $F_{(3, 168)}$ = 169.9 with p<0.0001 corresponding to 49.01% of the total variation. Response type $F_{(1, 168)}$ = 3.373 with p=0.0681 (0.3244% of total variation). Test period vs Cell type & genotype $F_{(3, 168)}$ = 5.906 with p=0.0007 (1.704% of total variation)	Tukey's response type classification: Decrease or Increase type, all p<0.0001 (these p values show correct classification approach). Tukey's comparing Z-score between response types (referred to colors in graph); [Halo] grey vs green p=0.6612, green vs grey p>0.9999. [SCH] grey vs green p=0.0068, green vs grey p=0.4366

Supplementary Table 3. Statistical details of Vglut2_ChR2 experiments (targeting PPN) with and without chemogenetic inhibition of CnF-Vglut2 neurons.

Group (n)	Figure/Result Claim	Outcome measured	Statistical approach	Descriptive report	Exact P and critical values
Vglut2_ChR2 =6 {sal}	**Fig.S3a**	Fraction of trials with locomotion detected	two-tailed, paired t-test	MEAN ± SEM > [baseline]= 1 ± 0.033; [saline]= 1 ± 0.022	p=0.3632; t=1, df=5
		Latency to initiate locomotion (s)	Wilcoxon matched-pairs signed rank test, two-tailed	MEAN ± SD > [baseline]= 2.451 ± 0.4928; [saline]= 2.987 ± 0.9719	p=0.0625, W=19 with rs(spearman) = 0.9429, p=0.0083
	**Fig.6b, line graph**	Distance moved (cm) per epoch [trials 6-20]	Two-way RM ANOVA		Laser $F_{(1.000, 5.000)}$ = 418.3 with p<0.0001
	**Fig.6b, inlet bars**	Average distance moved (cm) per epoch, before and after injection	Two-way RM ANOVA, Geisser-Greenhouse correction, Dunnett (vs 'pre')	MEAN > pre/laser/post> [baseline]= 37.60/62.67/16.54; [saline]= 24.88/55.52/9.678	Laser $F_{(1.266, 12.66)}$ = 55.29 with p<0.0001, accounts for 71.50% of the total variance. Dunnett [baseline] p=0.006 & 0.078, [saline] p<0.0001 & 0.023
Vglut2_ChR2 =10	**Fig.S3b**	Normalized distance moved per epoch (593nm laser)	Friedman test (RM), Dunn's (vs 'pre')	MEAN ± SD > [pre]= 100 ± 0; [laser]= 103.7 ± 33.16; [post]= 104.3 ± 48.71	Friedman test, T=3, S=10, Fstat=0.60 with p=0.8302; Dunn's, p>0.99 on both comparisons
Opto_CTRL =10 {sal}	**Fig.S3c**	Distance moved (cm) per epoch [trials 6-20]	Two-way RM ANOVA		Laser $F_{(1.000, 9.000)}$ = 0.004915 with p=0.9456
		Average distance moved (cm) per epoch, before and after injection	Two-way RM ANOVA, Geisser-Greenhouse correction, Dunnett (vs 'pre')	MEAN > pre/laser> [baseline]= 54.05/45.69; [saline]= 31.28/33.78	Laser $F_{(1.000, 9.000)}$ = 0.6878 with p=0.42, accounts for 1.11% of the total variance
Vglut2_ChR2 =10 {halo}	**Fig.6c, line graph**	Distance moved (cm) per epoch [trials 6-20]	Two-way RM ANOVA		Laser $F_{(1.000, 9.000)}$ = 18.56 with p=0.0020
	**Fig.6c, inlet bars**	Average distance moved (cm) per epoch, before and after injection	Two-way RM ANOVA, Geisser-Greenhouse correction, Dunnett (vs 'pre')	MEAN > pre/laser/post> [baseline]= 16.43/63.03/14.90; [halo]= 4.834/31.72/8.008	Laser $F_{(1.238, 22.29)}$ = 41.45 with p<0.0001, Dunnett [baseline] p=0.0008 & 0.903, [halo] p=0.0036 & 0.107
	**Fig.S3d**	Fraction of trials with locomotion detected	two-tailed, paired t-test	MEAN ± SEM > [baseline]= 0.98 ± 0.02; [halo]= 0.84 ± 0.06755	p=0.0310; t=2.553, df=9
		Latency to initiate locomotion (s)	Wilcoxon matched-pairs signed rank test, two-tailed	MEAN ± SD > [baseline]= 3.287 ± 1.030; [halo]= 4.895 ± 1.684	p=0.0488, W=39 with rs(spearman) = 0.030, p=0.4730
Vglut2_ChR2 =10 {SCH}	**Fig.6e, line graph**	Distance moved (cm) per epoch [trials 6-20]	Two-way RM ANOVA		Laser $F_{(1.000, 9.000)}$ = 20.11 with p=0.0015
	**Fig.6e, inlet bars**	Average distance moved (cm) per epoch, before and after injection	Two-way RM ANOVA, Geisser-Greenhouse correction, Dunnett (vs 'pre')	MEAN > pre/laser/post> [baseline]= 12.30/47.17/12.72; [SCH]= 2.205/18.58/2.504	Laser condition $F_{(1.424, 25.63)}$ = 50.14 with p<0.0001, Dunnett [baseline] p=0.0002 & 0.99, [halo] p=0.0028 & 0.69

Group (n)	Figure/Result Claim	Outcome measured	Statistical approach	Descriptive report	Exact P and critical values
	**Fig.S3e**	Fraction of trials with locomotion detected	two-tailed, paired t-test	MEAN ± SEM > [baseline]= 0.96 ± 0.02667; [SCH]= 0.7067 ± 0.06828	p=0.0081; t=3.682, df=9
		Latency to initiate locomotion (s)	Wilcoxon matched-pairs signed rank test, two-tailed	MEAN ± SD > [baseline]= 2.74 ± 1.206; [SCH]= 4.985 ± 1.887	p=0.0020, W=55 with rs(spearman) = 0.4061, p=0.1237
Vglut2_ChR2 =6 {sal}	**Fig.S4a**	Normalized distance moved (cm) per non-stimulus period	Wilcoxon matched-pairs signed rank test, one-tailed	MEAN ± SD > [1]= 100 ± 20.23; [2]= 80.51 ± 18.41. Median difference of 15.48%	Pairs=6, p=0.3438, W=-5
Vglut2_ChR2 =10 {halo}			Wilcoxon matched-pairs signed rank test, one-tailed	MEAN ± SD > [1]= 100 ± 19.81; [2]= 5.441 ± 0.7464. Median difference of 97.35%	Pairs=10, p=0.0010, W=-55
Vglut2_ChR2 =10 {SCH}			Wilcoxon matched-pairs signed rank test, one-tailed	MEAN ± SD > [1]= 100 ± 15.78; [2]= 17.83 ± 5.091. Median difference of 79.14%	Pairs=10, p=0.0010, W=-55
Vglut2_ChR2 =10 {halo}	**Fig.S4b**	Latency (s) to descent on bar test {no laser aid}	One sample Wilcoxon test, theoretical median 20s	MEAN ± SEM > 18.57 ± 0.4305	Discrepancy = 0, CI = -2.000 to 0.000 with actual confidence level = 97.72% with p=0.005
Vglut2_ChR2 =10 {SCH}			One sample Wilcoxon test, theoretical median 20s	MEAN ± SEM > 19.30 ± 0.3313	Discrepancy = 0, CI = 0 with actual confidence level = 95.72% with p=0.0078
Vglut2 _iD@CnF _ChR2@PPN =10 {sal vs CNO} **no opto**	**Fig.7b**	Corridor test, AUC	Area Under the Curve (AUC, per condition)	Saline = 123.5 ± 8.048 (SEM); CI = 107.8 to 139.3. CNO = 117.9 ± 5.419 (SEM); CI = 107.3 to 128.5	Saline peak at 0.9000s end reached in 3.6s. CNO peak at 0.9000s end reached in 4.5s
			two-tailed, paired t-test of AUC values per mouse	Difference between means = -6.740 ± 2.441 (SEM); CI = -12.26 to –1.219	p=0.0221; t=2.762, df=9
	**Fig.7c, left panel**	Corridor test, average speed (cm/s)	two-tailed, paired t-test	Difference between means = -18.48 ± 1.795 (SEM); CI = -22.54 to –14.42	p<0.0001; t=10.30, df=9, pairing correlation coefficient r=0.8813 with p=0.0004
	**Fig.7c, right panel**	Corridor test, max acceleration (m/s^2)	two-tailed, paired t-test	Difference between means = -12.44 ± 1.272 (SEM); CI = -15.31 to -9.559	p<0.0001; t=9.778, df=9, pairing correlation coefficient r=0.7629 with p=0.0051
Vglut2 _iD@CnF _ChR2@PPN =10 {sal vs CNO} **no opto**	**Fig.S5c, left panel**	Total distance moved (m)	two-tailed, paired t-test	MEAN ± SEM > CNO = 77.52 ± 8.342 and Difference between means = -25.87 ± 10.77 (SEM); CI = 50.23 to 1.508	p=0.0398; t=2.402, df=9, pairing correlation coefficient r=0.1451 with p=0.3446
	**Fig.S5c, right panel**	Distance moved (m) per time bin	Two-way RM ANOVA, Geisser Greenhouse correction, Bonferroni	ANOVA group effect $F_{(1, 18)}$ = 4.934 with p=0.0394 and 6.456% of the total variation. Group x Time $F_{(9, 162)}$ = 2.291 with p=0.0190 and 2.963% of the total variation	Time $F_{(2.739, 49.31)}$ = 33.83 with p<0.0001 and 43.75 of the total variation
	**Fig.S5d**	Percentage of time locomoting (%) (>2cm/s)	two-tailed, paired t-test	Difference between means = 6.181 ± 2.775 (SEM); CI = 0.3521 to 12.01	p=0.0389; t=2.228, df=18

Group (n)	Figure/Result Claim	Outcome measured	Statistical approach	Descriptive report	Exact P and critical values
	**Fig.S5e**	Percentage of time in each speed range when locomoting (%)	Two-way RM ANOVA, Geisser-Greenhouse correction, Bonferroni	Difference between means/speed range; [2-5]= 4.114, CI =0.09887 to 8.328, [5-10]= 1.756, CI = 0.4793 to 3.992, [10-20]=1.949, CI = 0.02296 to 3.921, [>20]=0.4091, CI = 1.246 to 2.064	Group x speed range $F_{(3, 54)}$ = 6.333 with p=0.0009 and representing only 0.8735% of the total variation.
Vglut2 _iD@CnF _ChR2@PPN = 10 {sal+CNO} **with opto**	**Fig.7d, line graph**	Distance moved (cm) per epoch [trials 6-20]	Two-way RM ANOVA		Laser $F_{(1.000, 9.000)}$ = 35.74 with p=0.0020
	**Fig.7d, inlet bars**	Average distance moved (cm) per epoch, before and after injection	Two-way RM ANOVA, Geisser-Greenhouse correction, Dunnett (vs 'pre')	MEAN > pre/laser/post> [baseline]= 21.82/71.15/11.44; [injected]= 20.06/55.33/11.82	Laser $F_{(1.538, 27.69)}$ = 82.36 with p<0.0001, Dunnett [baseline] p=0.0001 & 0.1174, [injected] p=0.0004 & 0.0402
	**Fig.7d vs 6b**	Average increase in distance ('laser'-'pre') [trials 6-20]	two-tailed, unpaired t-test	MEAN ± SEM > [**Fig.7d**]= 35.27 ± 5.899; [**Fig.6b**]= 30.63 ± 1.497	p=0.5624; t=0.5934, df=14
	**Fig.S5f-g, left panel**	Fraction of trials with locomotion detected	two-tailed, paired t-test	MEAN ± SEM > [baseline]= 0.94 ± 0.04269; [injected]= 0.92 ± 0.04532	p=0.7025; t=0.3943, df=9
		Latency to initiate locomotion (s)	Wilcoxon matched-pairs signed rank test, two-tailed	MEAN ± SD > [baseline]= 2.904 ± 0.6837; [injected]= 3.723 ± 0.7478	p=0.0488, W=39 with rs(spearman) = -0.2121, p=0.2801
Vglut2 _iD@CnF _ChR2@PPN =10 {halo+CNO} **with opto**	**Fig.7f, line graph**	Distance moved (cm) per epoch [trials 6-20]	Two-way RM ANOVA		Laser $F_{(1.000, 9.000)}$ = 61.68 with p<0.0001
	**Fig.7f, inlet bars**	Average distance moved (cm) per epoch, before and after injection	Two-way RM ANOVA, Geisser-Greenhouse correction, Dunnett (vs 'pre')	MEAN > pre/laser/post> [baseline]= 25.15/76.95/10.41; [injected]= 2.944/45.79/5.568	Laser $F_{(1.587, 28.56)}$ = 70.85 with p<0.0001, Dunnett [baseline] p=0.0003 & 0.1173, [injected] p<0.0001 & 0.1755
	**Fig. 7f vs 6c**	Average increase in distance ('laser'-'pre') [trials 6-20]	two-tailed, unpaired t-test	MEAN ± SEM > [**Fig.7f**]= 42.85 ± 5.456; [**Fig.6c**]= 26.88 ± 6.241	p=0.0700; t=1.926, df=18
	**Fig.S5f-g, middle panel**	Fraction of trials with locomotion detected	two-tailed, paired Student's t-test	MEAN ± SEM > [baseline]= 0.94 ± 0.06; [injected]= 0.9333 ± 0.03443	p=0.9059; t=0.1216, df=9
		Latency to initiate locomotion (s)	Wilcoxon matched-pairs signed rank test, two-tailed	MEAN ± SD > [baseline]= 3.001 ± 1.323; [injected]= 4.749 ± 1.84	p=0.0020, W=55 with rs(spearman) = 0.083, p=0.4788
Vglut2 _iD@CnF _ChR2@PPN =10 {SCH+CNO} **with opto**	**Fig.7h, line graph**	Distance moved (cm) per epoch [trials 6-20]	Two-way RM ANOVA		Laser $F_{(1.000, 9.000)}$ = 21.11 with p=0.0013
	**Fig.7h, inlet bars**	Average distance moved (cm) per epoch, before and after injection	Two-way RM ANOVA, Geisser-Greenhouse correction, Dunnett (vs 'pre')	MEAN > pre/laser/post> [baseline]= 21.79/59.61/5.386; [injected]= 3.627/40.01/6.595	Laser $F_{(1.354, 24.37)}$ = 40.94 with p<0.0001, Dunnett [baseline] p=0.0063 & 0.0286, [injected] p=0.0024 & 0.2583

Group (n)	Figure/Result Claim	Outcome measured	Statistical approach	Descriptive report	Exact P and critical values
	**Fig. 7h vs 6e**	Average increase in distance ('laser'-'pre') [trials 6-20]	two-tailed, unpaired t-test	MEAN ± SEM > [**Fig.7h**]= 36.38 ± 7.917; [**Fig.6e**]= 16.37 ± 3.651	p=0.0340; t=2.295, df=18
	**Fig.S5f-g, third panel**	Fraction of trials with locomotion detected	two-tailed, paired t-test	MEAN ± SEM > [baseline]= 0.96 ± 0.02667; [injected]= 0.8867 ± 0.05538	p=0.1456; t=1.593, df=9
		Latency to initiate locomotion (s)	Wilcoxon matched-pairs signed rank test, two-tailed	MEAN ± SD > [baseline]= 3.123 ± 1.144; [injected]= 4.590 ± 1.258	p=0.0371, W=41 with rs(spearman) = 0.2727, p=0.2241
Vglut2 _iD$_{@CnF}$ _ChR2$_{@PPN}$ = 10 {sal+CNO}	**Fig.S5e**	Normalized distance moved (cm) per non-stimulus period	Wilcoxon matched-pairs signed rank test, one-tailed	MEAN ± SD > [1]= 100 ± 37.33; [2]= 66.31 ± 35.23. Median difference of 27.43%	Pairs=10, p=0.0068, W=-47
=10 {halo+CNO}			Wilcoxon matched-pairs signed rank test, one-tailed	MEAN ± SD > [1]= 100 ± 49.97; [2]= 5.749 ± 4.987. Median difference of 72.26%	Pairs=10, p=0.0010, W=-55
=10 {SCH+CNO}			Wilcoxon matched-pairs signed rank test, one-tailed	MEAN ± SD > [1]= 100 ± 37.81; [2]= 7.564 ± 11.45. Median difference of 88.15%	Pairs=10, p=0.0010, W=-55
Vglut2 _iD$_{@CnF}$ _ChR2$_{@PPN}$ =10 {halo+CNO}	**Fig.S5f**	Latency (s) to descent on bar test {with and without laser aid}	Wilcoxon matched-pairs signed rank test, one-tailed	MEAN ± SEM > [laser off]17.67 ± 0.2650, [laser ON]3.967 ± 0.3199	Pairs=10, p=0.0010, W=-55 with median difference of 14.50
=10 {SCH+CNO}			Wilcoxon matched-pairs signed rank test, one tailed	MEAN ± SEM > [laser off]19.13 ± 0.2731, [laser ON]4.400 ± 0.4439	Pairs=10, p=0.0010, W=-55 with median difference of 14.50
Vglut2 _iD$_{@CnF}$ _ChR2$_{@PPN}$ =10 {CNO+opto}	**Fig.S5j**	Normalized distance moved per epoch (593nm laser)	Friedman test (RM), Dunn's (vs 'pre')	MEAN ± SD > [pre]= 100 ± 0; [laser]= 94.73 ± 31.39; [post]= 83.98 ± 36.66	Friedman test, T=3, S=10, Fstat=0.80 with p=0.7103; Dunn's, pre vs laser p>0.99 pre vs post p=0.7422

Supplementary Table 4. Statistical details of experiments involving chemogenetic activation of glutamatergic CnF neurons.

Group (n)	Figure/Result Claim	Outcome measured	Statistical approach	Descriptive report	Exact P and critical values
Vglut2_eD$_{@CnF}$ =5 {sal vs CNO}	**Fig.S6c, left panel**	Total distance moved (m)	two-tailed, paired t-test	MEAN ± SEM > CNO = 474.9 ± 63.66 and Difference between means = 286.2 ± 59.08 (SEM); CI = 122.1 to 450.2	p=0.0084; t=4.844, df=4
	**Fig.S6c, right panel**	Distance moved (m) per time bin	Two-way RM ANOVA, Geisser Greenhouse correction, Bonferroni	ANOVA group effect $F_{(1, 8)}$ = 18.94 with p=0 0024	Time x condition $F_{(9, 72)}$ = 8.750 with p<0.0001
	**Fig.S6d**	Percent of time locomoting (>2cm/s)	two-tailed, paired t-test	Difference between means = 0.4260 ± 6.135 (SEM); CI = -13.72 to 14.57	p=0.9463; t=0.06943, df=8
	**Fig.S6e**	Percent of time in each speed range when locomoting	Two-way RM ANOVA, Geisser-Greenhouse correction, Bonferroni	Difference between means/speed range; [2-5]= 17.50, CI =9.484 to 25.51, [5-10]= 11.44, CI = 1.743 to 21.14, [10-20]=-11.04, CI = -16.13 to -5.946, [>20]=17.19, CI = -28.95 to -6.848	Group x speed range $F_{(3, 24)}$ = 39.79 with p<0.0001 and representing 59.66% of the total variation
Sham^{CNO} =11, Vglut_eD$_{@PPN}^{CNO}$ =10, Vgat_eD$_{@PPN}^{CNO}$ =11, Vglut_eD$_{@CnF}^{Sal}$ =5, Vglut_eD$_{@CnF}^{CNO}$ =5, Vglut_iD$_{@CnF}^{Sal}$ =10, Vglut_iD$_{@CnF}^{CNO}$ =10	**Fig.S6f (all groups)**	Average speed when locomoting >20cm/s	Kruskal-Wallis test, Dunn's multiple comparisons vs Sham^{CNO}	p<0.0001, K-W=42.37	Adjusted p-values: Vglut_eD$_{@PPN}^{CNO}$ =0.0007, Vgat_eD$_{@PPN}^{CNO}$ =0.8356, Vglut_eD$_{@CnF}^{Sal}$ =0.5721, Vglut_eD$_{@CnF}^{CNO}$ =0.0008, Vglut_iD$_{@CnF}^{Sal}$ and Vglut_iD$_{@CnF}^{CNO}$ >0.9999
	**Fig.S6f (CnF vs PPN targeting)**		one-tailed, non-matched, Mann Whitney test	MEDIAN > CnF = 55.67; PPN = 34.70 cm/s. Median difference (Hodges-Lehmann) = 23.22	p=0.0003
Vglut2_eD$_{@CnF}$ =5 {sal vs CNO}	**Fig.S6g, left panel**	Stop bout frequency (count, Normalized)	two-tailed, paired t-test	Difference between means = -35.27 ± 10.34 (SEM); CI = -63.99 to -6.556	p=0.0270; t=3.410, df=4
	**Fig.S6g, right panel**	Stop bout duration (Normalized)	two-tailed, paired t-test	Difference between means = 67.20 ± 15.42 (SEM); CI = 24.39 to 110.0	p=0.0121; t=4.358, df=4
Vglut2_eD$_{@CnF}$ =5 {sal vs CNO}	**Fig.S6h (before-after)**	MSD ratio (cm/s/s) [see methods]	two-tailed, paired t-test	Difference between means = 56.89 ± 10.98 (SEM); CI = 26.40 to 87.38 whereas CI for the group mean under CNO is between 47.42 and 106.6 with Skewness of -1.095	p=0.0066; t=5.180, df=4, negative Skewness and high Kurtosis upon CNO injection indicates most locomotor events result in darting

Group (n)	Figure/Result Claim	Outcome measured	Statistical approach	Descriptive report	Exact P and critical values
				and Kurtosis of 1.392 (sal Skewness =0.1726, Kurtosis = -0.2585)	
Vglut_eD@CnF^{Sal} =5, Vglut_eD@CnF^{CNO} =5, Vglut_eD@PPN^{CNO} =10	**Fig.S6h (all groups)**		Kruskal-Wallis test, Dunn's multiple comparisons vs control Vglut_eD@CnF^{Sal}	p=0.0008, K-W=11.02	Adjusted p-values: Vglut_eD@PPN^{CNO} >0.9999, Vglut_eD@CnF^{CNO} =0.0083
Sham^{halo}= 8, Vglut2_eD@CnF$^{halo+CNO}$ =5	**Fig.S6i**	Distance moved (m) per time bin [before CNO]	Two-way RM ANOVA		Group effect $F_{(1, 11)}$ = 0.1135 with p=0.7425
		Distance moved (m) per time bin [after CNO]	Two-way RM ANOVA		Group effect $F_{(1, 11)}$ = 10.23 with p=0.0085
	**Fig.S6j**	Fold change of total distance moved per experimental period	two-tailed, unpaired t-test, Welch's correction	MEAN ± SEM> [Halo only] Sham = 1 ± 0.1830; Vglut = 0.8926 ± 0.2798; [after CNO] Sham = 1 ± 0.2731; Vglut = 4.987 ± 1.555	[Halo only]p=0.7569; t=0.3213, df=7.382 [after CNO] p=0.0307; t=2.525, df=4.248
Vglut2_eD@CnF$^{halo+CNO}$ =5 Wt$^{sal+sal}$ =5	**Fig.S6k**	Percent of time in each speed range when locomoting	Two-way RM ANOVA, Geisser-Greenhouse correction, Sidak's (vs WT$^{sal+sal}$)	Difference between means [speed range] WTxVglut; [2-5]= -22.28, [5-10]= -7.731, [10-20]= 9.257, [>20]= 20.76	Group x speed range $F_{(3, 24)}$ = 16.83 with p<0.0001
Vglut2_eD@CnF$^{halo+CNO}$ =5, Vglut2_eD@CnF^{Sal} =5	**Fig.S6n** graph shows only Halo+CNO, but stats is compared to baseline saline from panel S6h	CI of average MSD ratio (cm/s/s)	one-tailed, paired t-test	Difference between means 30.59±11.11. Halo mice with Skewness = -0.9955 and Kurtosis = 1.392	p=0.0256, t=2.754, df=4
Vglut2_eD@CnF =5 {Halo vs Halo+CNO}	**Fig.S6n**	Latency (s) to descent on bar test	two-tailed, Wilcoxon matched-pairs signed rank test	MEDIAN > Halo only = 18.67; after CNO = 12.00. Median difference = -6.667	p=0.0625, W=-15.00, Coefficient of variation upon repeated trials was of 5.095% (Halo only) and became 59.84% upon CNO

Supplementary Table 5. Statistical details of Vgat_eD PPN experiments.

Group (n)	Figure/Result Claim	Outcome measured	Statistical approach	Descriptive report	Exact P and critical values
$Sham^{CNO}$ =15	**Habituation**	Distance moved (m) per time bin	One-way RM ANOVA, Geisser Greenhouse correction, test for trend (time bin)	Linear trend $F_{(1, 126)}$ = 149.0 with p<0.0001. Slope = -1.395; SE of slope = -0.1143; CI of slope = -1.169 to -1.622 with p<0.0001	ANOVA Time effect: $F_{(4.453, 62.34)}$ = 16.87 with p<0.0001 and R squared = 0.5465
$Vgat\_eD^{CNO}$ =11	**maintained through 50min session**			Linear trend $F_{(1, 90)}$ = 25.80 with p<0.0001. Slope = -0.5528; SE of slope = -0.1088; CI of slope = -0.3366 to -0.7691 with p<0.0001	ANOVA time effect: $F_{(3.471, 34.71)}$ = 3.734 with p=0.0159 and R squared = 0.2719
$Sham^{CNO}$ =15, $Vgat\_eD^{CNO}$ =11	**Fig.8b, left panel**	Total distance moved (m)	two-tailed, unpaired t-test	MEAN ± SEM > Sham = 129.2 ± 9.805; Vgat = 217.5 ± 13.47 and Difference between means = 88.36 ± 16.24 (SEM); CI = 54.85 to 121.9	p<0.0001; t=5.442, df=24
	**Fig.8b, right panel**	Distance moved (m) per time bin	Two-way RM ANOVA, Geisser Greenhouse correction, Bonferroni	ANOVA group effect $F_{(1, 24)}$ = 27.41	Group x Time $F_{(9, 216)}$ = 3.203 with p=0.0012
	**Fig.8c**	Percentage of time locomoting (%) (>2cm/s)	two-tailed, unpaired t-test	Difference between means = 23.08 ± 3.094 (SEM); CI = 16.69 to 29.46	p<0.0001; t=7.458, df=24
	**Fig.8d**	Percentage of time in each speed range when locomoting (%)	Two-way RM ANOVA, Geisser-Greenhouse correction, Bonferroni	Difference between means/speed range; [2-5]= 5.689, CI =0.7431 to 10.63, [5-10]= -4.254, CI = -6.343 to -2.165, [10-20]= -2.501, CI = -5.443 to 0.4405, [>20]= 1.067, CI = -1.813 to 3.947	Group x speed range $F_{(3, 72)}$ = 9.713 with p<0.0001
$Sham^{CNO}$ =15, $Vglut2\_eD^{CNO}$ =10, $Vgat\_eD^{CNO}$ =11	**Fig.8f, left panel**	Stop bout frequency (count, Normalized)	Brown-Forsythe and Welch ANOVA, Dunnett (T3) (vs Sham)	Difference between means: Sham/Vglut = -25.31 ± 5.505 (SE); CI = -10.38 to -40.24 and Sham/Vgat = 42.29 ± 4.963 (SE); CI = 28.92 to 55.66	Brown-Forsythe ANOVA = $80.20_{(2.000, 31.69)}$ with p<0.0001; Welch's ANOVA = $102.4_{(2.000, 21.20)}$ with p<0.0001; Dunnett Vglut p=0.0003, Vgat p<0.0001
	**Fig.8f, right panel**	Stop bout duration (Normalized)	Brown-Forsythe and Welch ANOVA, Dunnett (T3) (vs Sham)	Difference between means: Sham/Vglut = -11.72 ± 11.63 (SE); CI = -44.08 to 20.64 and Sham/Vgat = 49.63 ± 7.264 (SE); CI = 29.54 to 69.73	Brown-Forsythe ANOVA = $21.16_{(2.000, 20.83)}$ with p<0.0001; Welch's ANOVA = $37.03_{(2.000, 17.33)}$ with p<0.0001; Dunnett Vglut p=0.5409, Vgat p<0.0001
$Sham^{halo}$ =8, $Vgat\_eD^{halo}$ =10	**Fig.9a**	Distance moved (m) per time bin [before CNO]	Two-way RM ANOVA		Group effect $F_{(1, 16)}$ = 1.723 with p=0.2079
		Distance moved (m) per time bin [after CNO]	Two-way RM ANOVA		Group effect $F_{(1, 16)}$ = 24.99 with p=0.0001
	**Fig.S7a**	Fold change of total distance moved per experimental period	two-tailed, unpaired t-test, Welch's correction	MEAN ± SEM> [Halo only] Sham = 1 ± 0.1830; Vgat = 1.411 ± 0.2376; [after CNO] Sham = 1 ± 0.2731; Vgat = 3.660 ± 0.4211	[Halo only]p=0.1903; t=1.369, df=15.73 [after CNO] p<0.0001; t=5.300, df=14.80
	**Halo induced bradykinesia**	Percentage of time in each speed range when		MEAN > Sham/Vgat > [2-5]= 60.75/71.14; [5-10]= 22.36/16.90; [10-20]=	Group x speed range $F_{(3, 48)}$ = 0.9591 with p=0.4197. Speed range alone $F_{(1.081,}$

Group (n)	Figure/Result Claim	Outcome measured	Statistical approach	Descriptive report	Exact P and critical values
		locomoting (%) [before CNO]		13.54/10.15; [>20]= 3.346/1.804	$_{17.30)}$ = 60.16 with p<0.0001
Sham$^{halo+CNO}$ =8, Vgat_eD$^{halo+CNO}$ =10, Wt$^{sal+sal}$ =5	**Fig.9b**	Percentage of time in each speed range when locomoting (%)	Two-way RM ANOVA, Geisser-Greenhouse correction, Dunnett (vs WT$^{sal+sal}$)	Difference between means [speed range] WTxSham/WTxVgat; [2-5]= -35.39/-34.14, [5-10]= 13.79/11.58, [10-20]= 15.79/15.55, [>20]= 5.814/7.007	Group x speed range $F_{(6, 60)}$ = 10.04 with p<0.0001
Sham$^{halo+CNO}$ =8, Vgat_eD$^{halo+CNO}$ =10	**Fig.9c**	Latency (s) to descent on bar test	two-tailed, non-matched, Mann Whitney test	MEDIAN > Sham = 18.67; Vgat = 2.167. Median difference (Hodges-Lehmann) = -16.67	p<0.0001 [calculated with ties among values]
Sham$^{SCH+CNO}$ =10, Vgat_eD$^{SCH+CNO}$ =10	**Fig.9d**	Distance moved (m) per time bin [before CNO]	Two-way RM ANOVA		Group effect $F_{(1, 18)}$ = 1.399 with p=0.2523
		Distance moved (m) per time bin [after CNO]	Two-way RM ANOVA		Group effect $F_{(1, 18)}$ = 0.05971 with p=0.8097
	**Fig.S7b**	Fold change of total distance moved per experimental period	two-tailed, unpaired t-test, Welch's correction	MEAN ± SEM> [SCH only] Sham = 1 ± 0.3648; Vgat = 0.5490 ± 0.1108; [after CNO] Sham = 1 ± 0.1583; Vgat = 1.051 ± 0.1339	[SCH only]p=0.2626; t=1.183, df=10.65 [after CNO] p=0.4049; t=0.2444, df=17.52
Sham$^{SCH+CNO}$ =10, Vgat_eD$^{SCH+CNO}$ =10, Wt$^{sal+sal}$ =5	**Fig.9e**	Percentage of time in each speed range when locomoting (%)	Two-way RM ANOVA, Geisser-Greenhouse correction, Dunnett (vs WT$^{sal+sal}$)	MEAN > WT/Sham/Vgat > [2-5]= 40.86/67.96/85.33; [5-10]= 29.17/19.89/11.11; [10-20]= 22.55/10.18/3.204; [>20]= 7.421/1.979/0.3565	Group x speed range $F_{(6, 66)}$ = 16.96 with p<0.0001
Sham$^{SCH+CNO}$ =10, Vgat_eD$^{SCH+CNO}$ =10	**Fig.9f**	Latency (s) to descent on bar test	two-tailed, non-matched, Mann Whitney test	MEDIAN > Sham = 18.67; Vgat = 3.333. Median difference (Hodges-Lehmann) = -14.67	p<0.0001 [calculated with ties among values]

Supplementary Table 6. Statistical details of Vgat_ChR2 PPN experiments.

Group (n)	Figure/Result Claim	Outcome measured	Statistical approach	Descriptive report	Exact P and critical values
Vgat_ChR2 =10 {sal}	**Fig.S8b**	Fraction of trials with locomotion detected	two-tailed, paired t-test	MEAN ± SEM > [baseline]= 0.9933 ± 0.006; [saline]= 1	p=0.3434; t=1, df=9
		Latency to initiate locomotion (s)	Wilcoxon matched-pairs signed rank test, two-tailed	MEAN ± SD > [baseline]= 1.690 ± 0.7719; [saline]= 1.879 ± 0.6350 Overall latency can vary between 1 to 4s.	p=0.2754, W=23 with rs(spearman) = 0.3576, p=0.1564
		Stop bout frequency (count, Normalized)	two-tailed, paired t-test	Difference between means = 15.34 ± 1.944 (SEM); CI = 10.94 to 19.74	p<0.0001; t=7.889, df=9 with pairing r = 0.7513
	**Fig.S8a, line graph**	Distance moved (cm) per epoch [trials 6-20]	Two-way RM ANOVA		Laser $F_{(1.000, 9.000)}$ = 14.96 with p=0.0038
	**Fig.S8a, inlet bars**	Average distance moved (cm) per epoch, before and after injection	Two-way RM ANOVA, Geisser-Greenhouse correction, Dunnett (vs 'pre')	MEAN > pre/laser/post> [baseline]= 59.59/68.77/68.14; [saline]= 60.53/76.71/70.73	Laser condition $F_{(1.903, 34.25)}$ = 10.96 with p=0.0003, accounts for 10.69% of the total variance. Dunnett [baseline] p=0.14 & 0.16, [saline] p=0.0051 & 0.0020
Vgat_ChR2 =10	**Fig.S9a**	Normalized distance moved per epoch (593 nm)	Friedman test (RM), Dunn's (vs 'pre')	MEAN ± SD > [pre]= 100 ± 0; [laser]= 86.16 ± 32.86; [post]= 87.24 ± 41.95	Friedman test, T=3, S=10, Fstat = 0.60 with p=0.8302; Dunn's, p>0.99 on both comparisons
Vgat_ChR2 =8 {halo}	**Fig.S8c, line graph**	Distance moved (cm) per epoch [trials 6-20]	Two-way RM ANOVA		Laser $F_{(1.000, 7.000)}$ = 15.93 with p=0.0052
	**Fig.S8c, inlet bars**	Average distance moved (cm) per epoch, before and after injection	Two-way RM ANOVA, Geisser-Greenhouse correction, Dunnett (vs 'pre')	MEAN > pre/laser/post> [baseline]=54.97/62.26/65.56; [halo]= 14.36/29.80/22.50	Laser condition $F_{(1.791, 25.07)}$ = 5.085 with p=0.0165. Dunnett [baseline] p=0.4678 & 0.3850, [halo] p=0.0095 & 0.0274
	**Fig.S8d**	Fraction of trials with locomotion detected	two-tailed, paired t-test	MEAN ± SEM > [baseline]= 1; [halo]= 0.7250 ± 0.05968	p=0.0025; t=4.608, df=7
		Latency to initiate locomotion (s)	Wilcoxon matched-pairs signed rank test, two-tailed	MEAN ± SD > [baseline]= 3.806 ± 1.415; [halo]= 6.709 ± 1.250	p=0.0020, W=55 with rs(spearman) = 0.6727, p=0.0195
Vgat_ChR2 =9 {SCH}	**Fig.S8e, line graph**	Distance moved (cm) per epoch [trials 6-20]	Two-way RM ANOVA		Laser $F_{(1.000, 8.000)}$ = 4.765 with p=0.0606
	**Fig.S8e, inlet bars**	Average distance moved (cm) per epoch, before and after injection	Two-way RM ANOVA, Geisser-Greenhouse correction, Dunnett (vs 'pre')	MEAN > pre/laser/post> [baseline]=45.56/62.44/66.32; [SCH]= 3.447/9.729/5.633	Laser condition $F_{(1.927, 30.84)}$ = 8.538 with p=0.0013. Dunnett [baseline] p=0.0523 & 0.0123, [halo] p=0.1049 & 0.3258
	**Fig.S8f**	Fraction of trials with locomotion detected	two-tailed, paired t-test	MEAN ± SEM > [baseline]= 0.9556 ± 0.02940; [SCH]= 0.2889 ± 0.09686	p=0.0001; t=7.071, df=8
		Latency to initiate locomotion (s)	Mann Whitney test, two-tailed	MEAN ± SD > [baseline]= 2.582 ± 0.9592; [SCH]= 5.563 ± 1.237	p=0.0007, U=2, ranks = 47, 89

Group (n)	Figure/Result Claim	Outcome measured	Statistical approach	Descriptive report	Exact P and critical values
Vgat_ChR2 =10 {sal}	Fig.S9d	Normalized distance moved (cm) per non-stimulus period	Wilcoxon matched-pairs signed rank test, one-tailed	MEAN ± SD > [1]= 100 ± 29.41; [2]= 90.68 ± 39.49, Median difference of 9.008%	Pairs=10, p=0.3477, W=-9
Vgat_ChR2 =8 {halo}			Wilcoxon matched-pairs signed rank test, one-tailed	MEAN ± SD > [1]= 100 ± 29.65; [2]= 1.619 ± 2.229. Median difference of 109.1%	Pairs=8, p=0.0039, W=-36
Vgat_ChR2 =9 {SCH}			Wilcoxon matched-pairs signed rank test, one-tailed	MEAN ± SD > [1]= 100 ± 37.20; [2]= 3.779 ± 10.35 Median difference of 99.40%	Pairs=9, p=0.0020, W=-45
Vgat_ChR2 =8 {halo}	Fig.S9e	Latency (s) to descent on bar test {no laser aid}	One sample Wilcoxon test, theoretical median 20s	MEAN ± SEM > 19.38 ± 0.2234	Discrepancy = 0, CI = 0 with actual confidence level = 97.34% with p=0.0625
Vgat_ChR2 =9 {SCH}			One sample Wilcoxon test, theoretical median 20s	MEAN ± SEM > 18.83 ± 0.4272	Discrepancy = 0, CI = -2.000 to 0 with actual confidence level = 95.73% with p=0.0039
Vgat_ChR2 =9 {SCH}	**re-test [20s laser]**	Average distance moved (cm) per epoch after injection	One-way RM ANOVA, Geisser-Greenhouse correction	MEAN > pre/laser/post> 2.724/14.52/13.77	Laser effect $F_{(1.857, 14.86)}$ = 7.174 with p=0.0074

Supplementary Table 7. Statistical details of motor proficiency upon PPN stimulation.

Group (n)	Figure/Result Claim	Outcome measured	Statistical approach	Descriptive report	Exact P and critical values
**Kinematics with DeepLabCut** Sham^{CNO} =11, Vglut_eD^{CNO} =10, Vgat_eD^{CNO} =11, Vglut_eD$^{halo+CNO}$ =8, Vglut_eD$^{SCH+CNO}$ =6, Vgat_eD$^{halo+CNO}$ =6, Vgat_eD$^{SCH+CNO}$ =5	**Fig.S10a**	Speed range profile of all video segments extracted from the Open Field (% of total time)	Descriptive	Speed range with highest preference (cm/s): Sham^{CNO} and Vglut_eD^{CNO} =10-15, All other groups 5-10cm/s	Vgat_eD$^{halo+CNO}$ and Vgat_eD$^{SCH+CNO}$ show Bradykinesia with high percent of time at 0-5cm/s [30.63 and 28.47%, respectively]
	**Fig.S10b**	Speed average (cm/s) within videos used for Kinematics analysis	One-way ANOVA, Dunnett's vs Sham^{CNO}	Difference between group and Sham^{CNO}, means, CI; [Vglut_eD^{CNO}]= -3.2, -5.1 to -1.3, [Vgat_eD^{CNO}]= 1.6, -0.2 to 3.5, [Vglut_eD$^{halo+CNO}$]=3.2, 1.2 to 5.2, [Vglut_eD$^{SCH+CNO}$]=1.1, -1.1 to 3.2, [Vgat_eD$^{halo+CNO}$]= 4.2, 1.9 to 6.4, [Vgat_eD$^{SCH+CNO}$]=6.3, 4 to 8.6	$F_{(6, 50)}$ = 27.14 with p<0.0001. Dunnett's p= 0.0002; 0.1041; 0.0006; 0.6240; <0.0001; <0.0001, in figure order.
	**Fig.S10c**	Step frequency (Hz)	One-way ANOVA, Dunnett's vs Sham^{CNO}	Max difference to Sham^{CNO} was found for Vgat$^{SCH+CNO}$ = 1.181±0.1680Hz (SE)	$F_{(6, 50)}$ = 13.27 with p<0.0001. Dunnett's p= >0.9999; 0.9997; 0.8890; 0.1337; 0.0001; <0.0001 in figure order
	**Fig.10b**	Knee and Ankle travel (normalized to step cycle)		Line starts at paw touchdown and is plotted with Locally Weighted Scatterplot Smoothing of 10points, full curve made on 80 sequential points and based on all strides performed by each mouse	Shadow zones with 95% Confidence interval
		Percentage of stance time (paw in contact with floor, normalized to step cycle)	One-way ANOVA, Tukey's	Max difference between two groups was Vgat^{CNO} to Vgat$^{SCH+CNO}$ = -10.67 ±2.231% (SE)	$F_{(6, 50)}$ = 6.473 with p<0.0001. Tukey's p= 0.0208; 0.0003; 0.0376 in figure report order
	**Fig.10c**	Amplitude Knee angle (normalized to step cycle)	One-way ANOVA, Tukey's	The average variation of each mouse is calculated without applying any smoothing	$F_{(6, 50)}$ = 4.672 with p=0.0008. Tukey's p= 0.0004; 0.0020; 0.0096 in figure report order
		Amplitude Ankle angle (normalized to step cycle)	One-way ANOVA, Tukey's	The average variation of each mouse is calculated without applying any smoothing	$F_{(6, 50)}$ = 13.02 with p<0.0001. Tukey's p<0.0001 for all reported in figure
	**Fig.10e**	Shank Travel (normalized to step cycle and max angle)	Step cycles individually normalized to max Shank angle=1, minimum=0.	Line starts at paw touchdown and is plotted with Locally Weighted Scatterplot Smoothing of 10points, a full curve made on 80 sequential points	Shadow zones with 95% Confidence interval.
		Area Under Curve analysis (AUC)	Peak position report as % of step cycle	Peak X position: [Sham^{CNO}]= 79.75, [Vglut_eD^{CNO}]= 79.75, [Vgat_eD^{CNO}]= 79.75, [Vglut_eD$^{halo+CNO}$]= 79.75, [Vglut_eD$^{SCH+CNO}$]= 69.62, [Vgat_eD$^{halo+CNO}$]= 81.01, [Vgat_eD$^{SCH+CNO}$]= 89.87	

Group (n)	Figure/Result Claim	Outcome measured	Statistical approach	Descriptive report	Exact P and critical values
Ladder test Vglut2^{wt} -CTRL =5 no opto, Vglut2_ChR2 =4, Vgat_ChR2 =4/3 {halo **or** SCH} with opto	**Fig.10e, left panel**	Percentage of functional steps (%)	Kruskal-Wallis test, Dunn's multiple comparisons vs CTRL	p=0.0674, K-W=8.758, 5 groups compared, overall, no effect	Adjusted p-values: Vglut_ChR2^{halo} =0.5898, Vglut_ChR2^{SCH} =0.4610, Vgat_ChR2 halo and SCH both >0.9999
	**Fig.10e, right panel**	Percentage of steps targeting same rug as forelimb (%)	Kruskal-Wallis test, Dunn's multiple comparisons vs CTRL	p=0.0233, K-W=11.30, 5 groups compared	Adjusted p-values: Vglut_ChR2^{halo} =0.7897, Vglut_ChR2^{SCH} =0.4214, Vgat_ChR2^{halo} >0.9999, Vgat_ChR2^{halo} =0.0064
	**Average speed when locomoting**	Average speed (cm/s)	One-way ANOVA, Dunnett's vs CTRL	Mean and range; [CTRL]= 7.753, CI =6.3 to 9.2, [Vglut^{halo}]= 7.233, CI = 5.4 to 9.1, [Vglut^{SCH}]= 7.39, CI = 6.6 to 8.2, [Vgat^{halo}]=6.2, CI = 4.8 to 7.5, [Vgat^{SCH}]= 5.4, CI = 2.4 to 8.3	Treatment $F_{(4, 15)}$ = 3.332 with p=0.0385 and r^2=0.4705. Dunnett's Vglut: [halo]=0.8715, [SCH]=0.9592, Vgat [halo]=0.1210, [SCH]=0.0218
Obstacle corridor Vglut2 _iD@CnF _ChR2@PPN = 10 {sal vs CNO+SCH+Opto}	**Fig.S10f, left panel**	Number of full crosses (n)	two-tailed, paired t-test	Difference between means = -1.200 ± 1.954 (SEM); CI = -5.62 to 3.22	p=0.5543, t=0.6142, df=9
	**Fig.S10f, right panel**	Total distance covered (m)	two-tailed, paired t-test	Difference between means = 1.207 ± 1.724 (SEM); CI = -2.693 to 5.108	p=0.5015, t=0.7002, df=9
	**Fig.S10g**	Average speed when crossing (cm/s)	two-tailed, paired t-test	Difference between means = 1.067 ± 4.169 (SEM); CI = -1.915 to 4.050	p=0.4390, t=0.8096, df=9
Obstacle corridor Vglut2 _iD@CnF _ChR2@PPN = 10 {sal vs CNO+SCH+Opto}	**Fig.S10h**	Average speed per obstacle (cm/s)	Two-way RM ANOVA, Holm-Sidak's	Obstacle vs Session accounts to only 0.3871 % of total variation whereas obstacles account for 33.75 [$F_{(2, 27)}$= 13.81 with p<0.0001] and individual variation for 32.98% [$F_{(27, 27)}$= 1.005 with p=0.4953]	Obstacle vs Session effect $F_{(2, 27)}$= 0.1591 with p=0.8537, [Rod] p=0.9753, [Slalom] p=0.9753, [Stairs] p=0.9753
	**Object Occupancy (saline)**	Index (%), percentage of time spend per obstacle, corrected by obstacle length	One-way RM ANOVA, Tukey's	Mean differences: [Rod vs Slalom]=21.28 CI=6.7 to 35.76, [Rod vs Stairs]= 15.03 CI= 0.54 to 29.52, [Slalom vs Stairs]= -6.245 CI= -20.73 to 8.24	Obstacle effect $F_{(2, 18)}$= 7.423 with p=0.0045, [Rod vs Slalom] p=0.0040, [Rod vs Stairs] p=0.0413, [Slalom vs Stairs] p=0.5262
	**Object Occupancy (between sessions)**		Two-way RM ANOVA, Sidak's (obstacle per session)	Difference between means [Rod]=-4.921 CI -18 to -28, [Slalom]= -3.844 CI -27 to 19, [Stairs]= -1.077 CI -24 to 22	Obstacle vs Session effect $F_{(2, 18)}$= 0.2492 with p=0.7821, [Rod] p=0.9312, [Slalom] p=0.9652, [Stairs] p=0.9992
Obstacle corridor Vglut2 _iD@CnF	**Fig.S10h**	Latency (s) to descent on bar test {with or without laser}	Two-way RM ANOVA, Bonferroni	Difference between means [Start vs End]= -1.433 Ci -2.64 to -0.23, [Start vs End+laser]= 12.60 CI 11.36 to 13.81	Condition $F_{(2, 40)}$ = 443.5 with p<0.0001, Bonferroni [Start vs End] p=0.0172,

Group (n)	Figure/Result Claim	Outcome measured	Statistical approach	Descriptive report	Exact P and critical values
_ChR2@PPN = 10 {CNO+SCH }					[Start vs End+laser] p<0.0001

Supplementary Table 8. Mouse cohorts used on this study. Open Field (OF), Kinematics (K). (*) Exclusions from test round due to lack of immobility on the Bar test. Optical stimulation (blue text, 473nm; Orange, 593nm).

Mouse cohort	Groups	Behavior Batteries	Anatomy
**Cohort 1**<br>Chemogenetic PPN activation	5 WT [no surgery]	5 OF^{sal}<br>5 OF$^{sal+sal}$, Bar test	-
	15 Sham<br>[5*Vglut2*^{wt}_retroCre, 5*Vgat*^{cre}_mCherry, 5Vglut2^{cre}_mCherry]	15 OF^{CNO} [11 K]<br>8 OF$^{halo+CNO}$, Bar test**<br>10 OF$^{SCH+CNO}$, Bar test	Check
	10 Vglut2^{cre}	10 OF^{CNO} [10 K]<br>10 OF$^{halo+CNO}$, Bar test [8 K]<br>10 OF$^{SCH+CNO}$, Bar test [6 K]	Check, images shown
	11 Vgat^{cre}	11 OF^{CNO}[11 K]<br>10 OF$^{halo+CNO}$, Bar test [6 K]<br>10 OF$^{SCH+CNO}$, Bar test [5 K]	Check, images shown
**Cohort 2**<br>Chemogenetic CnF activation	5 Vglut2^{cre}	5 OF^{sal}<br>5 OF^{CNO}<br>5 OF$^{halo+CNO}$, Bar test	Coronal Map
**Cohort 3**<br>Optogenetic PPN activation	10 Ctrl<br>[5*Vglut2*^{cre}_mCherry, 5*Vgat*^{wt}_retroCre]	10 OF^{sal}	Check
	10 Vglut2^{cre}	6 OF^{sal}<br>10 OF^{halo}, Bar test<br>10 OF^{SCH}, Bar test<br>10 Laser wavelength<br>4 Ladder^{halo}<br>4 Ladder^{SCH}	Sagittal Map
	10 Vgat^{cre}	10 OF^{sal}<br>8 OF^{halo}, Bar test**<br>9 OF^{SCH}, Bar test*<br>9 OF re-test^{SCH}, Bar test*<br>10 Laser wavelength<br>4 Ladder^{halo}<br>4 Ladder^{SCH}	Sagittal Map
	5 Ctrl [Vglut2^{wt}]	5 Ladder^{sal}	Check
**Cohort 4**<br>Optogenetic activation of PPN with concomitant CnF inhibition	10 Vglut2^{cre}	10 Corridor^{sal}<br>10 Corridor^{CNO}<br>10 OF^{sal}<br>10 OF^{CNO}<br>10 OF$^{sal+CNO}$<br>10 OF$^{halo+CNO}$, Bar test<br>10 OF$^{SCH+CNO}$, Bar test<br>10 Laser wavelength^{CNO}<br>10 Obstacle^{sal}<br>10 Obstacle$^{SCH+CNO}$, Bar test	Coronal Map
	8 WT [Drd1^{wt}]	Corridor exposure only	-
**Cohort 5**<br>Calcium imaging of STR or PPN	4 Vglut2^{cre}	4 OF^{halo}, Bar test<br>4 OF^{SCH}, Bar test	Coronal implant map
	4 Drd1^{cre}	4 OF^{halo}, Bar test<br>4 OF^{SCH}, Bar test	Coronal implant map
**Cohort 6**<br>Chemogenetic activation of PPN for c-Fos analysis	5 Vglut2^{cre}	5 OF^{CNO}<br>5 CNO-Perfusion	Coronal Map
	5 Vgat^{cre}	5 OF^{CNO}<br>5 CNO-Perfusion	Coronal Map
	2 WT [Vgat^{wt}]	2 OF^{CNO}<br>2 CNO-Perfusion	Coronal Map

Description of Movie Files

Movie 1. Optogenetic activation of caudal glutamatergic PPN rescues parkinsonian phenotype.
Adult Vglut2^{cre} mice following the injection of a Cre-dependent AAV-DIO-ChR2 virus in the caudal PPN and chronic implantation of an optical fibre for optogenetic activation of glutamatergic neurons. Video starts showing individual mice prior to drug injection. Upon injection of drugs that antagonize dopamine signalling (haloperidol and SCH23390) mice show akinesia and difficulty to initiate movement (Bar test). Approximately 30min after drug injection, parkinsonian mice were placed in a series of environments to demonstrate motor recovery upon PPN stimulation of caudal glutamatergic neurons. Note how motor proficiency over different environments is maintained.

Light activation of transfected neurons was applied at 40Hz (10s total duration with 10ms square pulses, using 473nm light at 2-3.5mW, connector tip). Video is shown as a montage to allow observation of response variety amongst mice. Recordings were done with infrared reporter light, placed on the upper left corner of each panel showing when the laser is activated (see methods, Bar test). [1min 5s, 223MB]

Movie 2. Activation of caudal GABAergic PPN neurons in parkinsonian mice.
Adult Vgat^{cre} mice following the injection of a Cre-dependent AAV-DIO-ChR2 virus in the caudal PPN and chronic implantation of an optical fibre for optogenetic activation of GABAergic neurons. Approximately 30min after injection of drugs that antagonize dopamine signalling (haloperidol and SCH23390) mice show akinesia and difficulty to initiate movement. Approximately 30min after drug injection, parkinsonian mice were placed in a series of environments to demonstrate motor performance upon stimulation.

Light activation of transfected neurons was applied at 40Hz (10s total duration with 10ms square pulses, using 473nm light at 2-3.5mW, connector tip). Video is shown as a montage to allow observation of response variety amongst mice. Recordings were done with infrared reporter light, placed on the upper left corner of each panel showing when the laser is activated (see methods, Bar test). [50s, 175MB]

Both videos are: 720x576 frames, 28809kbps, 25fps .avi
