# Supplementary material for: Targeted activation of midbrain neurons restores locomotor function in mouse models of parkinsonism
Source: Nat Commun. 2022 Jan 26;13:504. doi: 10.1038/s41467-022-28075-4 (PMC8791953; doi:10.1038/s41467-022-28075-4)
Supplement: Supplementary file 2 — Reporting Summary [file 41467_2022_28075_MOESM2_ESM.pdf]

nature research

Corresponding author(s): Ole Kiehn

Last updated by author(s): Dec 20, 2021

Reporting Summary

Nature Research wishes to improve the reproducibility of the work that we publish. This form provides structure for consistency and transparency in reporting. For further information on Nature Research policies, see our Editorial Policies and the Editorial Policy Checklist.

Statistics

For all statistical analyses, confirm that the following items are present in the figure legend, table legend, main text, or Methods section.

n/a	Confirmed	
☐	☒	The exact sample size (*n*) for each experimental group/condition, given as a discrete number and unit of measurement
☐	☒	A statement on whether measurements were taken from distinct samples or whether the same sample was measured repeatedly
☐	☒	The statistical test(s) used AND whether they are one- or two-sided<br>*Only common tests should be described solely by name; describe more complex techniques in the Methods section.*
☐	☒	A description of all covariates tested
☐	☒	A description of any assumptions or corrections, such as tests of normality and adjustment for multiple comparisons
☐	☒	A full description of the statistical parameters including central tendency (e.g. means) or other basic estimates (e.g. regression coefficient) AND variation (e.g. standard deviation) or associated estimates of uncertainty (e.g. confidence intervals)
☐	☒	For null hypothesis testing, the test statistic (e.g. *F*, *t*, *r*) with confidence intervals, effect sizes, degrees of freedom and *P* value noted<br>*Give P values as exact values whenever suitable.*
☒	☐	For Bayesian analysis, information on the choice of priors and Markov chain Monte Carlo settings
☒	☐	For hierarchical and complex designs, identification of the appropriate level for tests and full reporting of outcomes
☐	☒	Estimates of effect sizes (e.g. Cohen's *d*, Pearson's *r*), indicating how they were calculated

Our web collection on statistics for biologists contains articles on many of the points above.

Software and code

Policy information about availability of computer code

Data collection	For behavioral analysis, mice were tracked using Ethovision 15.0 software (Noldus). In vivo calcium imaging data was acquired using Inscopix Data acquisition (DAQ) box and Inscopix nVoke acquisition system (v2.0), software (IDAS, v1.3.1). Microscope imaging was performed on ZEISS Axio Scan equipped with Colibri light system (tiles, 10X) or LSM900 (confocal 20-40X) and detailed description given on methods. Limb Kinematics were analyzed with DeepLabCut (v.2.2b8) to obtain tracks based on digital markers, the training of a ResNet-50-based neural network is detailed on methods.
Data analysis	Behavior data was preprocessed within the Ethovision software (v.15.0). Calcium Imaging raw data processing was performed using the software package from Inscopix (Inscopix Data Processing Software, v1.3.0.2723). Microscopy images where processed using the Apeer image processing platform (a recent initiative by ZEISS to offer customizable solutions to the microscopy community), ZEN 3.2 blue (free software), Zen Pro (v.3.0) and ImageJ (v.1.53J) plugins. Kinematics was preprocessed with Deeplabcut (v2.2b8) and its available toolbox, and Ladder test tracking done with TSE Motion Video Analysis Software (v.9.2.2).<br>Subsequent post-processing analysis were performed using JMP software (v14, SAS Institute Inc), statistics & graphs were done with either JMP or GraphPad Prism (v.8.4.1) and images were adjusted for print with Adobe Illustrator (v.2020).

For manuscripts utilizing custom algorithms or software that are central to the research but not yet described in published literature, software must be made available to editors and reviewers. We strongly encourage code deposition in a community repository (e.g. GitHub). See the Nature Research guidelines for submitting code & software for further information.

Data

Policy information about availability of data

All manuscripts must include a data availability statement. This statement should provide the following information, where applicable:

- Accession codes, unique identifiers, or web links for publicly available datasets
- A list of figures that have associated raw data
- A description of any restrictions on data availability

The manuscript does not contain data analyzed with custom built scripts. The scripts used rely on available plugins within the softwares (described above). Analysis sequences are described in methods. Source data file is available with this article and contains the data used to build all figures. Raw video files/images are available upon reasonable request.

Field-specific reporting

Please select the one below that is the best fit for your research. If you are not sure, read the appropriate sections before making your selection.

☒ Life sciences ☐ Behavioural & social sciences ☐ Ecological, evolutionary & environmental sciences

For a reference copy of the document with all sections, see nature.com/documents/nr-reporting-summary-flat.pdf

Life sciences study design

All studies must disclose on these points even when the disclosure is negative.

Sample size	Effect size was unknown beforehand, and the minimum group size was initially defined based on the assumption that the mean of control and treated groups would need to differ by 20% (δ) to be considered a biologically meaningful effect, whereas SD would remain within a 10% (σ) margin regardless of treatment. σ margin was selected based on the distance moved in the open field by healthy-WT mice when handled by the experimenter. With α = 0.05 and power level of 95%, we estimated a minimum group size to be 7. Those same parameters with adjusted SD to 13% (σ) resulted in group sizes of 11 mice. Thus, we defined, a priori, that the minimum group size for a behavioral experimental round should be set to 7 mice/group and that a second batch of animals would be generated for the completion of groups aiming at n = 10. As a result, for behavioral analysis, the article contains groups of 10 mice and there are instances in which merging of batches resulted in higher group number, in which case all animals were included in the data set.<br>Mice from different genotypes, that performed multiple testings (behavioral batteries) are described as belonging to a cohort. Cohorts are clearly stated on methods section. Due to the length of each experimental round, tests were performed in sequential days, until all experimental mice had been tested. In such instances groups where balanced so to be represented in each experimental day (parallel testing). The exact n per group is indicated in every experiment (legend or within the figure), and always present within the supplementary statistical tables.
Data exclusions	All exclusion events are indicated within the article supplementary figures and criteria for those decisions is clearly described in methods. In optogenetic experiments the Bar test was considered as an exclusion criterion for Open Field data analysis whenever mice did not score high akinesia defined as an average latency of 15s latency to descent (during laser-OFF). This exclusion criteria was defined a priory with the aim to include in our analysis only animals that showed a high degree of parkinsonian-state.
Replication	We ran 2 replication batches which are not included in the manuscript. First, with 7 Vglut2-cre mice injected with AAV-DIO-ChR2 for Open Field - Haloperidol. This test was performed on a different field size/shape and results were in line with data sets presented. Second, a batch with 10 WT mice injected with AAV-CamKII-ChR2 for Open Field - Haloperidol and SCH23390. Again, results were in line with the data presented in the article.
Randomization	Allocation of animals into groups was done following the minimization approach (ARRIVE guidelines) and took into consideration the sex, age bracket and initial weight bracket (nuisance variable control). Control groups were selected by minimizing the imbalance across factors. Moreover, pharmacological experiments and optogenetic experiments where set so that outcome measures could, in most part, be assessed with each subject/group as its own control (pre-post experimental interference approach).
Blinding	During allocation and conduct of experiment: the experimenter was not blind to the conditions of the experiment (content of injection/ subject group) because experimental design required injection specificity and group composition needed to be balanced through the hours of the day. During outcome assessment and data analysis we introduced blinding steps which are described in the methods section. All analysis were done with automated tracking and scoring of behaviors.

Reporting for specific materials, systems and methods

We require information from authors about some types of materials, experimental systems and methods used in many studies. Here, indicate whether each material, system or method listed is relevant to your study. If you are not sure if a list item applies to your research, read the appropriate section before selecting a response.

Materials & experimental systems

n/a	Involved in the study
	☒ Antibodies
☒	☐ Eukaryotic cell lines
☒	☐ Palaeontology and archaeology
	☒ Animals and other organisms
☒	☐ Human research participants
☒	☐ Clinical data
☒	☐ Dual use research of concern

Methods

n/a	Involved in the study
☒	☐ ChIP-seq
☒	☐ Flow cytometry
☒	☐ MRI-based neuroimaging

Antibodies

Antibodies used

anti-DsRed made in rabbit, Takara #632496; anti-GFP made in chicken, Abcam #13970; anti-c-Fos made in rabbit, Cell Signaling # 9F6, Deep red Neurotrace [640/660], Invitrogen #N21483; Alexa-568 anti rabbit, Invitrogen #A10042; Alexa-488 anti chicken, Invitrogen #A11039; Alexa-405 anti rabbit, Invitrogen #A31556.

Validation

-NeuroTrace is selective for the Nissl substance characteristic of neurons and provides more sensitivity than traditional histological dyes like toluidine blue or cresyl violet.
-Anti-GFP [Chicken polyclonal to GFP, UniProtKB - P42212 (GFP_AEQVI)]: This antibody was used to facilitate the identification of post fixed GCaMP6s positive neurons - GCaMP is created from a fusion containing the green fluorescent protein (GFP).
-Anti-DsRed can be used to detect red fluorescent proteins such as mCherry and td-Tomato.
-Anti-cFos detects endogenous levels of total c-Fos protein. The antibody does not cross-react with other Fos proteins, including FosB, FRA1 and FRA2.
All above mentioned antibodies are validated for use on immunolabeling on the supplier website and show no cross-reaction with other endogenous proteins in the rodent nervous system.
We observed no Anti-GFP or Anti-DsRed labeling on WT mice (primary antibody control experiment was performed). Furthermore, we tested Anti-GFP and Anti-DsRed for specificity by staining mice with viral-mCherry expression with anti-GFP and mice with viral-Citrine expression with anti-DsRed. In both approaches no cross labeling was observed.
For secondary antibodies, parallel experiments were run, in each staining round, by either eliminating the primary antibody step or replacing it with serum of the same species. No nonspecific binding was observed. These control staining experiments are also stated in methods.

Animals and other organisms

Policy information about studies involving animals; ARRIVE guidelines recommended for reporting animal research

Laboratory animals

Genetic mouse strains: heterozygous Vglut2-cre, Vgat-cre, and Drd1-cre (Tg(Drd1a-cre)150Gsat; FK150Gsat). Strains were kept under C57Bl6j background. Male and female mice were used (approx. 1:1). Ages 8-10 weeks at experiment start, maximum of 5 months old at experimental end (calcium imaging exception; 7.5 months old, but see methods). The specificity of these mouse lines has been described in previous work, citations to those articles is within the methods section "experimental subjects".

Wild animals

none.

Field-collected samples

none.

Ethics oversight

All animal experiments and procedures were in accordance with the EU Directive 20110/63/EU and approved by the Danish Animal Inspectorate (Dyreforsøgstilsynet, Ethical permit: 2017-15-0201-01172, P21-326).

Note that full information on the approval of the study protocol must also be provided in the manuscript.
