# Supplementary material for: Targeted activation of midbrain neurons restores locomotor function in mouse models of parkinsonism
Source: Nat Commun. 2022 Jan 26;13:504. doi: 10.1038/s41467-022-28075-4 (PMC8791953; doi:10.1038/s41467-022-28075-4)
Supplement: Supplementary file 3 — Description of Additional Supplementary Files [file 41467_2022_28075_MOESM3_ESM.pdf]

Title: Supplementary Movie 1.

Description: Optogenetic activation of caudal glutamatergic PPN rescues parkinsonian phenotype. Adult Vglut2cre mice following the injection of a Cre-dependent AAV-DIO-ChR2 virus in the caudal PPN and chronic implantation of an optical fibre for optogenetic activation of glutamatergic neurons. Video starts showing individual mice prior to drug injection. Upon injection of drugs that antagonize dopamine signalling (haloperidol and SCH23390) mice show akinesia and difficulty to initiate movement (Bar test). Approximately 30min after drug injection, parkinsonian mice were placed in a series of environments to demonstrate motor recovery upon PPN stimulation of caudal glutamatergic neurons. Note how motor proficiency over different environments is maintained. Light activation of transfected neurons was applied at 40Hz (10s total duration with 10ms square pulses, using 473nm light at 2-3.5mW, connector tip). Video is shown as a montage to allow observation of response variety amongst mice. Recordings were done with infrared reporter light, placed on the upper left corner of each panel showing when the laser is activated (see methods, Bar test). [1min 5s, 223MB]

Title: Supplementary Movie 2.

Description: Activation of caudal GABAergic PPN neurons in parkinsonian mice. Adult Vgatcre mice following the injection of a Cre-dependent AAV-DIO-ChR2 virus in the caudal PPN and chronic implantation of an optical fibre for optogenetic activation of GABAergic neurons. Approximately 30min after injection of drugs that antagonize dopamine signalling (haloperidol and SCH23390) mice show akinesia and difficulty to initiate movement. Approximately 30min after drug injection, parkinsonian mice were placed in a series of environments to demonstrate motor performance upon stimulation.

Light activation of transfected neurons was applied at 40Hz (10s total duration with 10ms square pulses, using 473nm light at 2-3.5mW, connector tip). Video is shown as a montage to allow observation of response variety amongst mice. Recordings were done with infrared reporter light, placed on the upper left corner of each panel showing when the laser is activated (see methods, Bar test). [50s, 175MB].
